# Development and validation of a simple, cost-effective competitive allele-specific PCR assay for largescale screening and detection of Fec^B^ mutation in sheep

**DOI:** 10.1371/journal.pone.0337392

**Published:** 2025-12-02

**Authors:** Kathiravan Periasamy, Rudolf Pichler, Saravanan Ramasamy, Rajesh Kumar Somasundaram, Muladno Basar, Atanaska Teneva, Amadou Traore, Arnaud S. R. Tapsoba, Mohammed O. Faruque, Seyed Abbas Rafat, Tanveer Hussain

**Affiliations:** 1 Animal Production and Health Laboratory, Joint FAO/IAEA Centre of Nuclear Techniques in Food and Agriculture, Department of Nuclear Sciences and Applications, International Atomic Energy Agency, Vienna, Austria; 2 Indian Council of Agricultural Research-National Research Centre on Mithun, Medziphema, Nagaland, India,; 3 Tamil Nadu Veterinary and Animal Sciences University, Chennai, India; 4 Department of Animal Sciences, Bogor Agricultural University, Bogor, Indonesia; 5 University of Forestry, Sofia, Bulgaria; 6 Laboratoire de Biologie et Santé animales (LaBioSA), Institut de l’Environnement et de Recherches Agricoles (INERA), Ouagadougou, Burkina Faso; 7 Department of Animal Breeding and Genetics, Bangladesh Agricultural University, Mymensingh, Bangladesh; 8 Department of Animal Science, Faculty of Agricultural Sciences, University of Tabriz, Tabriz, Iran; 9 Animal Genomics & Biodiversity Group, Department of Biological Sciences, Virtual University of Pakistan, Islamabad, Pakistan; West Bengal University of Animal and Fishery Sciences, INDIA

## Abstract

The Booroola fecundity (Fec^B^) gene, a mutation in the bone morphogenetic protein receptor-1B (BMPR1B), is known to increase ovulation rate and litter size in sheep. Efficient and accurate, large-scale detection of this single nucleotide polymorphism (SNP) is critical for marker assisted introgression/selection programs for genetic improvement of prolificacy. This study aimed to develop and validate a robust, cost-effective, and high-throughput genotyping assay for detecting the FecB mutation across diverse sheep populations globally. A competitive allele-specific PCR (KASP) assay targeting the A746G nucleotide substitution in BMPR1B gene was designed and tested on a diverse panel of 224 individuals across 22 sheep breeds and validated against direct sequencing. The KASP genotyping results showed 100% concordance with sequence data, with clear fluorescence-based discrimination of homozygous wild-type Fec^++^ (AA), heterozygous Fec^B+^ (AG), and homozygous mutant Fec^BB^ (GG) genotypes. The assay was compatible across three real-time PCR platforms, BioRad CFX96, Roche LightCycler 480 II, and ABI QuantStudio 6 demonstrating reproducible genotyping and cross-platform interoperability. Subsequently, the KASP assay was applied to a global screening of 1471 sheep from 47 breeds in 14 countries for further validation of the assay. The mutation was not observed in the sheep samples investigated from Africa, Europe, Latin America, and West Asia, whereas it was polymorphic or fixed in certain populations from South (India and Bangladesh) and Southeast Asia (Indonesia). Notably, native Bangladeshi sheep exhibited high frequencies of the Fec^B^ allele, with some populations (e.g., Bangladesh Central) nearing fixation, reflecting possible selection for high prolificacy. In conclusion, the validated KASP assay offers a powerful, scalable tool for the rapid genotyping of the Fec^B^ mutation, enabling efficient screening and incorporation of prolificacy traits in sheep breeding programs worldwide.

## Introduction

Prolificacy, defined by the ability to produce multiple offsprings per birth, is a critical trait with significant implications on the efficiency and productivity of sheep farming. Higher prolificacy with more number of lambs born per ewe/year can significantly enhance overall flock productivity [1]. For smallholder farmers, who often operate with limited resources and land, higher prolificacy translates directly into increased flock size and enhanced meat production per breeding cycle [2]. This increased productivity can significantly boost household income and food security, allowing smallholders to maximize returns on their investment in feed, labour and veterinary care. Additionally, sheep breeds with high prolificacy can contribute to rapid genetic improvements within flocks, as more offspring provide more selection opportunities for desirable traits.

The Fec^B^ (Booroola) mutation in sheep has garnered significant attention due to its profound impact on prolificacy. Fec^B^ mutation is a single nucleotide polymorphism in the BMPR1B (Bone Morphogenetic Protein Receptor 1B) gene that plays a critical role in regulating ovulation rates and litter size [3]. The substitution (A746G) in exon 8 of the BMPR1B gene leads to an amino acid change (Q249R) that alters receptor activity and disrupts the normal inhibitory signalling pathway of bone morphogenetic proteins in granulosa cells. This functional alteration induces maturation of ovarian follicles by increasing the sensitivity of the follicles to Follicle Stimulating Hormone (FSH), thus resulting in higher ovulation rate and litter size. The weighted mean advantage of ewes carrying one copy of Fec^B^ mutation was estimated to be + 1.3 (range +0.8 to +2.0) for ovulation rate and +0.7 (range +0.4 to +1.3) for litter size across different production systems and varying recipient genetic background [1]. As the use of sheep carrying the FecB gene offers a substantial increase in fecundity in one generation, crossing experiments have been undertaken in several countries to evaluate and introgress FecB into other breeds [1]. Large-scale introgression and marker-assisted selection efforts have successfully incorporated the FecB allele into low-prolific breeds demonstrating substantial gains in lambing percentage and reproductive efficiency under diverse production systems. This mutation’s ability to significantly boost fecundity makes it a valuable genetic marker for breeding programs aimed at improving reproductive efficiency in various sheep breeds.

Fec^B^ mutation was initially found in the Booroola Merino sheep, a separate line of ewes selected for high litter size in Australia [4,5] and later hypothesized to have originated from Garole sheep in India [6]. The mutation was subsequently reported in several other breeds including Garut [7] and fat tailed sheep of Indonesia [8], Hu and Han sheep of China [9]; and Kendrapada sheep of India [10]. Among these, Fec^B^ mutation has been found to be almost fixed in Garole sheep breed [11,12]. The incorporation of the Fec^B^ mutation into breeding programs involves various strategies, including crossbreeding and within-breed selection. Crossbreeding native breeds with those carrying the Fec^B^ mutation can introduce this advantageous trait into populations with lower fecundity.

Few genotyping methods have been employed to detect Fec^B^ mutation in sheep [13–16] of which the forced PCR-RFLP (Polymerase Chain Reaction-Restriction Fragment Length Polymorphism) method [17] is the most widely used. This technique amplifies the target region within BMPR1B gene and uses restriction enzyme (Ava II) to identify the mutation. Although this method is reliable and specific for detecting Fec^B^ mutation but time-consuming and relatively more expensive for large-scale screening required for selection/introgression programs. Real-Time PCR based genotyping methods offer a faster, efficient and cost-effective alternative and can process large numbers of samples simultaneously, making it ideal for high-throughput applications. The Kompetitive Allele-Specific PCR (KASP) assay (LGC BioSearch Technologies, Hoddesdon, UK) is a fluorescence-based, endpoint PCR technique widely used for detecting single nucleotide polymorphisms (SNPs). KASP employs two allele-specific forward primers and one common reverse primer, each tagged with unique fluorescent labels. During amplification, competitive binding of primers ensures allele-specific extension, and fluorescence resonance energy transfer (FRET) enables precise genotypic discrimination. The chemistry is homogeneous, requiring no post-PCR processing, and is adaptable to high-throughput formats. Owing to its accuracy, cost-effectiveness, and scalability, KASP is a good alternative to traditional genotyping methods such as PCR-RFLP and sequencing. Hence, the present study was undertaken with the following objectives: (i) develop a competitive allele-specific PCR based assay to detect Fec^B^ mutation in sheep (ii) validate the assay through targeted sequencing approach and optimize the assay across different real time PCR platforms and (iii) evaluate the assay performance for high throughput screening and perform preliminary assessment on distribution of Fec^B^ alleles in diverse sheep breeds/populations located across different geographical regions.

## Materials and methods

### Animal sampling and ethics statement

Blood samples were collected from various sheep breeds/ populations located across Asia, Africa, Europe and Latin America. A total of 1471 sheep belonging to 47 breeds and located across 14 countries were utilized for the study (Table 1). Blood sampling was performed by qualified veterinarians from different sheep breeds following the standard guidelines and good animal handling practice. The blood samples were collected as part of routine veterinary surveillance and informed written consent was obtained from animal owners for the usage of a part of those samples for DNA extraction and analysis in this study. The necessary clearance from Institutional Biosafety and Ethical Committee, Tamil Nadu Veterinary and Animal Sciences University (USO No. 50066/G2/2012-PROC.NO. 7699) and BioScience Ethical Committee, Virtual University of Pakistan (Ref.No. ECS/2021/23) was obtained for the study. All efforts were made to ensure ethical and safe handling of the animal subjects during the process of blood sample collection.

**Table 1:** Genotype count and frequency of BMPR1B (Fec^B^) mutations across sheep populations worldwide.

Region	Country	Breed name	Breed code	N	Genotype count			Genotype frequency			Allele frequency	
					AA	AG	GG	AA	AG	GG	A	G
Africa	Burkina Faso	Djallonke	DJA	99	99	0	0	1	0	0	1	0
		Mossi	MOS	76	76	0	0	1	0	0	1	0
		Sahelian	SHL	16	16	0	0	1	0	0	1	0
	Ethiopia	Menz	MEN	90	90	0	0	1	0	0	1	0
		Washera	WAS	7	7	0	0	1	0	0	1	0
	Sudan	Abrag	ABR	31	31	0	0	1	0	0	1	0
		Ashgar	ASG	6	6	0	0	1	0	0	1	0
		Beja	BEJ	16	16	0	0	1	0	0	1	0
		Hammari	HMR	31	31	0	0	1	0	0	1	0
		Kabashi	KAB	17	17	0	0	1	0	0	1	0
Europe	Austria	KrainerSteinschaf	KSF	135	135	0	0	1	0	0	1	0
		Mouflon	MUF	6	6	0	0	1	0	0	1	0
		Texel	TEX	18	18	0	0	1	0	0	1	0
	Bulgaria	Karakachanska	KAR	14	14	0	0	1	0	0	1	0
		Shumenska	SHM	15	15	0	0	1	0	0	1	0
		Westplaminska	WEP	16	16	0	0	1	0	0	1	0
	Germany	Bergschaf	BER	22	22	0	0	1	0	0	1	0
Latin America	Argentina	Corriedale	COR	7	7	0	0	1	0	0	1	0
	Peru	Junin	JNN	39	39	0	0	1	0	0	1	0
South Asia	Bangladesh	Bangladesh Central	BGC	22	1	0	21	0.045	0	0.955	0.045	0.955
		Bangladesh East	BGE	20	9	10	1	0.450	0.500	0.050	0.700	0.300
		Bangladesh North	BGN	17	5	6	6	0.294	0.353	0.353	0.471	0.529
		Garole	GAR	21	1	6	14	0.048	0.286	0.667	0.190	0.810
	India	GaroleXSandyno cross	GAS	7	0	7	0	0	1	0	0.5	0.5
		Garole	IGR	7	0	0	7	0	0	1	0	1
		Kilakarsal	KKL	50	50	0	0	1	0	0	1	0
		Mecheri	MEC	58	58	0	0	1	0	0	1	0
		Madras Red	MRS	60	60	0	0	1	0	0	1	0
		NARI Composite	NAR	11	0	1	10	0	0.091	0.909	0.045	0.955
		Nellore	NEL	44	44	0	0	1	0	0	1	0
		Nilgiri	NIL	8	0	8	0	0	1	0	0.5	0.5
		Nilgiri X Sandyno cross	NIS	11	0	1	10	0	0.091	0.909	0.045	0.955
		Pattanam	PAT	54	54	0	0	1	0	0	1	0
		Sandyno	SAN	6	2	4	0	0.333	0.667	0	0.667	0.333
		Vembur	VEM	42	42	0	0	1	0	0	1	0
	Pakistan	Kachi	KAC	15	15	0	0	1	0	0	1	0
		Kajli	KAJ	13	13	0	0	1	0	0	1	0
		Karakul	KUL	17	17	0	0	1	0	0	1	0
		Thalli	THA	17	17	0	0	1	0	0	1	0
South East Asia	Indonesia	Indonesian Fat Tailed	IFT	17	3	3	11	0.176	0.176	0.647	0.265	0.735
		Indonesian Thin Tailed	ITT	93	60	22	11	0.645	0.237	0.118	0.763	0.237
West Asia	Iran	Ghezel	GHE	70	70	0	0	1	0	0	1	0
		Makui	MAK	19	19	0	0	1	0	0	1	0
		Moghani	MOG	19	19	0	0	1	0	0	1	0
		Shal	SHA	23	23	0	0	1	0	0	1	0
	Iraq	Awassi	AWA	22	22	0	0	1	0	0	1	0
		Hamdani	HAM	47	47	0	0	1	0	0	1	0

### Inclusivity in global research

Additional information regarding the ethical, cultural, and scientific considerations specific to inclusivity in global research is included in the S1 file.

### DNA extraction and quality control

Genomic DNA was extracted using MasterPure DNA Purification Kit (Biozym, Illumina Inc, USA), procured and distributed by Animal Production and Health Laboratory, Joint FAO/IAEA Centre to respective collaborating institutions as part of the International Atomic Energy Agency (IAEA) – Coordinated Research Project. DNA was extracted at the laboratories of the collaborating institutions and subjected to quality control using Nanodrop 2000 spectrophotometer (ThermoScientific, USA) to estimate concentration, 260/280 and 260/230 ratios. DNA samples were shipped to Animal Production and Health Laboratory, IAEA Laboratories, Seibersdorf, Austria, subjected to secondary quality and quantity check using Nanodrop 2000 spectrophotometer and stored at -20^o^C until further processing.

### Primer design, PCR amplification and sequencing BMPR1B gene

The Fec^B^ mutation alters the Bone Morphogenetic Protein Receptor Type-1B (BMPR1B) gene on sheep chromosome 6. This mutation, characterized by an A746G nucleotide change in the BMPR1B gene’s coding region, results in a glutamine-to-arginine substitution at position 249 (Q249R) of the amino acid sequence. A partial region (30011796−30212793) within the reference sequence of sheep chromosome 6 (NC_056059.1) under the NCBI-GenBank accession number CM028709.1 was used as template for designing oligonucleotide primers. The primers were designed to amplify partial BMPR1B gene using Primer 3 v.4.1.0 [18]. The size of the amplicon was 870 bp and the sequence of forward and reverse primers were as follows: BMPR1BSeq1-F 5’-GATCGAACCCGAGTCTCTTG-3’ and BMPR1BSeq1-R 5’-AACAGGCACACACTCCATGA-3’. Polymerase chain reaction was performed in a total reaction volume of 40μl with the following cycling conditions: initial denaturation at 95˚C for 15 min followed by 30 cycles of 95˚C for 45s; 57˚C for 30s; 72˚C for 30s with final extension at 72˚C for 10 min. The PCR composition included 4µl of template DNA (10ng/µl), 1.2µl each of forward and reverse primers (5pmol/µl), 4µl of dNTP (2mM) mix (Invitrogen, Thermo Fisher Scientific Corporation, CA, USA), 0.3µl of Taq polymerase (Solis Biodyne, Tartu, Estonia), 4µl of 10X PCR buffer and 25.3µl of PCR water. PCR amplifications were confirmed by running 7µl of PCR product mixed with 2 µl of gel loading dye mixed with Gel Red on 1.5% agarose gel at a constant voltage 120 V for 60 min in 1X TAE buffer. Purified PCR products were sequenced from both ends using Big Dye Terminator Cycle Sequencing Kit (Applied Biosystems, U.S.A) on an automated Genetic Analyzer ABI 3100 (Applied Biosystems, U.S.A). A total of 224 sheep (S2 File) belonging to 22 breeds located across diverse geographic regions were amplified and sequenced. Sequences generated from both ends were edited using Codon Code Aligner version 11.0.2 and secondary peaks were called to ascertain the Fec^B^ mutation. The forward and reverse sequences were assembled to generate contigs using Codon Code Aligner.

### Design and validation of competitive allele-specific PCR assay for FecB genotyping

The competitive allele-specific PCR (KASPar) assay for genotyping Fec^B^ mutation was custom designed (LGC BioSearch Technologies, Hoddesdon, UK) and assay conditions for genotyping on different real time PCR platforms were optimized at FAO/IAEA Laboratories, International Atomic Energy, Seibersdorf, Austria. The sequences of allele-specific forward primers were 5’- TTCATGCCTCATCAACACCGTCT-3’ and 5’-CATGCCTCATCAACACCGTCC-3’ respectively while the sequence of common reverse primer was 5’- CAGCTGGTTCCGAGAGACAGAAATA-3’. The KASP assay was performed in a total reaction volume of 10μl with a composition of 2µl of template DNA (5ng/μl), 0.138µl of KASP primer mix (two forward and one common reverse primers), 5µl of 2X KASP Reaction buffer and 2.862µl of PCR water. The thermal cycling conditions for end point genotyping included a touch down protocol as follows: Step1: 94°C for 15 minutes; Step2: 94°C for 20 seconds; Step3: 61°C for 60 seconds; Step4: Drop – 0.6°C/per cycle and repeat steps 2–3 for nine times (a total of 10 cycles) achieving the annealing temperature of 55°C; Step5: 94°C for 20 seconds; Step6: 55°C for 60 seconds; Repeat steps 5–6 for 25 times (a total of 26 cycles). The endpoint allele discrimination module incorporated within the BioRad CFX96 (BioRad, USA) was utilized for genotyping. If individuals within each genotype cluster are scattered, an additional thermal recycling was performed as follows: Step1: 94°C for 20 seconds; Step2: 55°C for 60 seconds; repeat steps 1–2 twice (a total of 3 cycles). The accuracy of allele calling was confirmed by comparing the genotypes derived from KASPar assay with the available sequence data on individuals from diverse sheep breeds.

### PCR-RFLP genotyping

A total of 159 sheep belonging to different breeds were genotyped using PCR-RFLP method for further comparison and validation of results from competitive allele-specific PCR assay. PCR amplification was performed using the primers 5’-CCAGAGGACAATAGCAAAGCAAA-3’ (TestF2) and 5’- CAAGATGTTTTCATGCCTCATCAACACGGTC-3’ (TestR15) as described elsewhere [6]. Polymerase chain reaction was performed in a total reaction volume of 40μl with the following cycling conditions: initial denaturation at 95˚C for 15 min followed by 30 cycles of 95˚C for 30s; 60˚C for 30s; 72˚C for 30s with final extension at 72˚C for 10 min. The PCR composition included 4µl of template DNA (10ng/µl), 1.2µl each of forward and reverse primers (5pmol/uL), 4µl of dNTP (2mM) mix (Invitrogen, Thermo Fisher Scientific Corporation, CA, USA), 0.3µl of Taq polymerase (Solis Biodyne, Tartu, Estonia), 4µl of 10X PCR buffer and 25.3µl of PCR water. The resulting PCR product with size of 190-base pair (bp) was then digested using restriction enzyme *Ava*II (New England Biolabs, Beverly, MA) and separated on a 2.5% agarose gel electrophoresis. The mutant FecB allele (G) digested to yield a 160-bp fragment while the wild type allele yielded (A) an undigested fragment size of 190-bp, the band patterns of which were used for assigning genotypes.

### Cross-platform compatibility testing

All standard experiments were performed on CFX96^TM^ touch real time PCR detection system (Bio-Rad, CA, USA). However, to evaluate the applicability of the assay across different real time PCR platforms, the same thermal cycling conditions were tested on Quantstudio^TM^6 Flex (Applied Biosystems, Life Technologies, Thermo Fisher Scientific Corporation, CA, USA) and LightCycler 480 II (Roche, Basel, Switzerland). To obtain optimal genotyping results, KASP buffer with standard ROX was used for BioRad CFX96 while the KASP buffer with low ROX was used for ABI Quantstudio 6 Flex and Roche LightCycler 480 II. A subset of 47 samples representing all the three genotypes (homozygous Fec^BB^, heterozygous Fec^B+^ and homozygous wild type Fec^++^) including no-template and positive controls were utilized for this purpose. Additionally, the competitive allele-specific PCR was performed in two steps: (i) touch down polymerase chain reaction was performed on a conventional PCR machine (BioRad C1000) and (ii) the resultant PCR amplicons were transferred to BioRad CFX96 real time PCR detection system to perform the end point measurement and genotyping.

## Results

### Design and performance of KASP assay for genotyping FecB mutation

**Fig 1:**
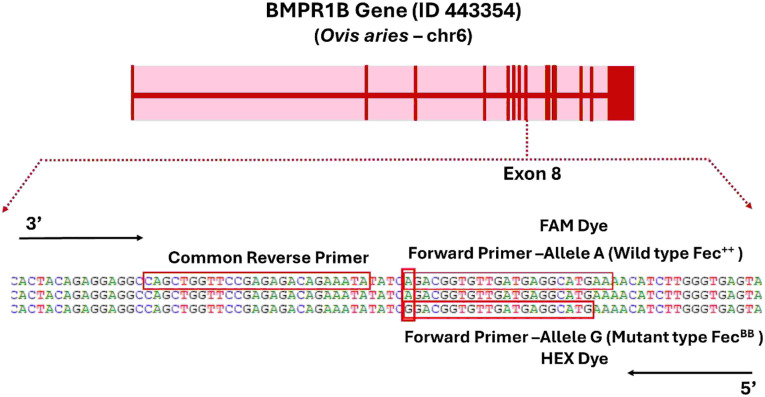
Illustration of KASP assay design for genotyping Fec^B^ mutation in sheep.

The competitive allele-specific PCR (KASP) assay (LGC BioSearch Technologies, Hoddesdon, UK) offers a simple, cost-effective method for SNP genotyping, with potential application in large-scale screening of genetic mutations and marker-assisted selection/introgression programs. In this study, we developed a KASP genotyping assay for detecting Fec^B^ mutation (A746G) in the BMPR1B gene associated with enhanced prolificacy in sheep. Genotypes for the wild type Fec^++^ (AA), homozygous Fec^BB^ (GG), and heterozygous Fec^B+^ (AG) were determined using fluorescence-based endpoint detection. The KASP assay mix contains three assay-specific non-labelled oligos: two allele-specific forward primers and one common reverse primer (Fig 1). All the three primers were designed using Primer Digital (https://primerdigital.com/tools/kasp.html) online tool by submitting the flanking sequence containing the site of mutation (single nucleotide polymorphic locus) in square parenthesis. To design the KASP assay, two forward primers were synthesized, each allele-specific and tagged with proprietary tail sequences (LGC BioSearch Technologies, Hoddesdon, UK) enabling detection through fluorescence resonance energy transfer (FRET) chemistry. One forward primer specific to the mutant Fec^B^ (G) allele was tagged with a HEX™ dye-compatible tail, while the forward primer for the wild-type Fec^+^ (A) allele was labelled with a FAM™ dye-compatible tail. A single common reverse primer was used in both reactions (Fig 1). Primer design was guided by multiple sequence alignment of BMPR1B gene regions across several sheep breeds to ensure the flanking sequences were conserved and to avoid off-target amplification. To perform the genotyping, a touch-down PCR protocol was applied to improve specificity.

**Fig 2:**
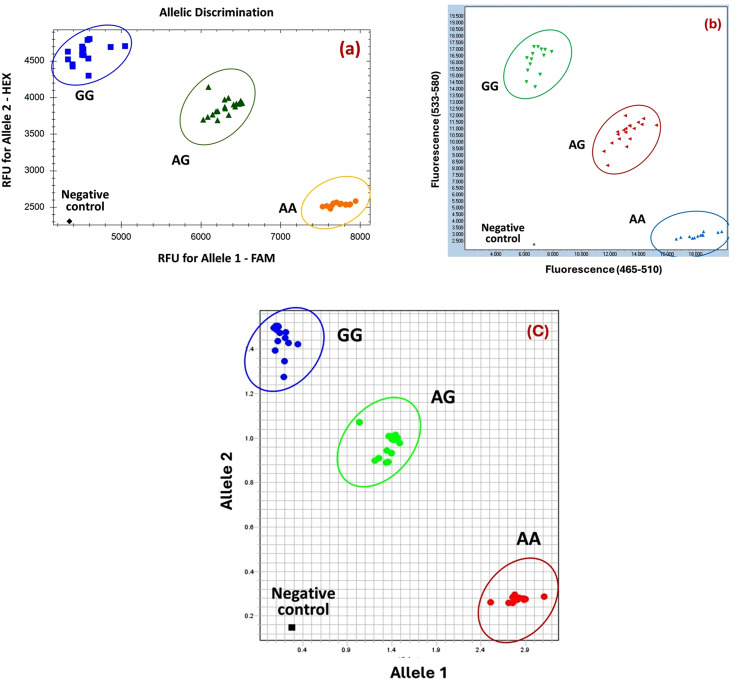
Clustering of BMPR1B (FecB) genotypes across three real time PCR platforms (a) BioRad CFX (b) Roche Light Cycler LC480 and (c) ABI Quantstudio 6.

The endpoint allele discrimination module incorporated within the BioRad CFX96 (BioRad, USA) was utilized for calling the genotypes based on fluorescent intensity recorded for each of the two alleles. Samples homozygous for the mutant Fec^B^ allele (GG) displayed relatively high HEX fluorescence, those homozygous for the wild-type Fec^+^ allele (AA) displayed relatively high FAM fluorescence, and heterozygotes (AG) showed both. The emission data of all the samples in the plate were plotted in X and Y axis respectively for each allele and the genotypes were called based on distinct clustering. As shown in Fig 2, distinct and tightly clustered groups were observed for all three genotypes, confirming the precision of the assay. The bottom-right cluster (high FAM, low HEX) corresponded to homozygous wild type Fec^+^(AA) individuals, indicating exclusive amplification of the mutant allele. The top-left cluster (high HEX, low FAM) represented homozygous Fec^B^ mutant (GG) individuals, signifying successful amplification of only the wild-type allele. The central diagonal cluster, with balanced intermediate fluorescence from both dyes, represented heterozygous (AG) individuals, reflecting co-amplification of both alleles [19]. This tight and well-separated clustering confirmed the specificity and efficiency of the allele-specific primers. One of the important features of KASP assay chemistry is its fluorescence-based signal amplification mechanism, which relies on the interaction between allele-specific primers and universal FRET cassette oligos. Upon successful primer extension, fluorescent signals corresponding to either FAM™ or HEX™ dyes are released and accumulate in a linear, amplification-dependent manner. The signal intensity is proportional to allele-specific amplicons, enabling highly sensitive detection even at low template concentrations. Hence, if genotype clusters remained diffuse after 26 cycles, recycling the amplicons for additional three cycles helped in enhancing signal amplification, distinct clustering of genotypes and accuracy of genotyping [20].

### Validation of KASP assay for genotyping FecB mutation

The validation of the KASP assay was conducted by (i) using positive and no template controls and (ii) comparing the results of KASP genotyping with PCR-RFLP and sequence data. Positive controls for each of the three genotypes (AA, AG, GG) and no template control (NTC) were included in each run along with the test samples. The positive controls served as reference points to validate clustering positions and genotype calls across different assay plates. Each control consistently clustered within the expected genotype group, reinforcing the reliability of allele discrimination. Furthermore, as expected, NTCs displayed no measurable fluorescence signal in either channel (FAM/HEX) and appeared in the bottom-left corner of the scatter plot, distinctly separated from all genotyped samples. Their proper placement confirmed the absence of background noise or cross-reactivity and affirmed the technical integrity of the assay. Positive controls provided genotype confirmation and clustering reference points, while negative controls validated the assay specificity and contamination-free status [19,21,22]. Together, they ensured that observed sample clusters reflected true allelic differences rather than technical artifacts.

The second level of validation encompassed the concordance of KASP genotypes with PCR-RFLP genotype (S3 File) and sequence data at *BMPR1B* locus. Among 159 sheep samples genotyped using both PCR-RFLP (S4 File) and competitive allele-specific PCR assays, one sample (FBVN-5) showed discordance (S2 File). Specifically, the PCR-RFLP analysis indicated a homozygous GG genotype, while the competitive allele-specific PCR assay classified the sample as heterozygous AG. Subsequent Sanger sequencing of the sample confirmed the AG genotype, thereby validating the result of KASP assay. Further, a total of 224 samples representing a wide diversity of sheep breeds distributed across different geographical regions were utilized for comparison of KASP genotype and sequence results. The sequence data of the partial BMPR1B gene generated in this study are available at NCBI-GenBank accession numbers OR767427-OR767650. Across all samples tested, a complete concordance was observed between the KASP genotypes and sequence results (S2 File). Each of the AA, AG, and GG genotypes determined by sequencing was correctly and consistently matched by the corresponding KASP genotypes, indicating a 100% genotype calling accuracy. This agreement across all three genotypic classes demonstrated the robustness of the KASP assay in distinguishing not only homozygous alleles but also the heterozygous state, which is often more prone to misclassification [23,24]. Notably, the KASP assay exhibited no allelic dropout, miscalls, or discrepancies when cross-validated against sequencing data. Further, the assay maintained high level of accuracy irrespective of genetic background or sample origin, suggesting that its performance is stable and not confounded by potential sequence variability flanking the mutation site [25]. It also demonstrated a high signal-to-noise ratio, with clear clustering of genotypes and minimal ambiguity, as inferred from the absolute match with sequencing results.

### Cross-platform comparison and compatibility

The compatibility and accuracy of the genotyping assay was evaluated across three widely used real-time PCR systems: BioRad CFX96, Roche LightCycler 480 II, and ABI QuantStudio 6. A total of 47 samples representing sheep from diverse populations (including Bangladeshi, Indonesian, and composite flocks) were analysed in all three platforms. Instrument-specific PCR composition protocols were optimized to harmonize KASP amplification efficiency, including standard touchdown PCR conditions and appropriate fluorescence plate read settings. Comparative performance was assessed using amplification reliability, clustering consistency and genotype concordance. The genotyping output was consistent across platforms with a concordance of 100%, encompassing homozygous wild-type Fec^++^ (AA), heterozygous Fec^B+^ (AG), and homozygous mutant Fec^BB^ (GG) individuals (Table 2). For instance, samples such as IFT-9H2, IFT-12N04, and FBVN-25 exhibited consistent GG genotypes, while IFT-13H39, IFT-14H40, and BGBGN20D were uniformly typed as AA across systems. Similarly, heterozygous individuals (e.g., IFT-10N03, ITT-21ET01, and BGBGN09D) were classified as AG with no discrepancies (Table 2). Only one instance of partial dropout was observed (sample FBVN-28 on Roche Light Cycler LC480 II), attributed to a non-determined call possibly due to optical interference or insufficient fluorescence intensity. Moreover, allele discrimination plots generated on all three systems showed well-resolved genotype clusters with no overlap or misclassification, demonstrating the robustness of the assay (Fig 2a-c). The absence of genotypic discordance across platforms and the demonstrated interoperability ensures that data generated from one platform can be reliably compared or pooled with data from other real time PCR systems. Furthermore, the cross-platform compatibility affirms the suitability of assay for high-throughput applications across different laboratories, including national/regional scale genotyping projects.

**Table 2:** Comparative results of BMPR1B (Fec^B^) genotyping across three real time PCR platforms.

Well No.	Sample Name	BioRad CFX96	Roche LC480 II	ABI Quantstudio 6	Sequence
A01	IFT-9H2	GG	GG	GG	GG
B01	IFT-10N03	AG	AG	AG	AG
C01	IFT-11H4	AG	AG	AG	AG
D01	IFT-12N04	GG	GG	GG	GG
E01	IFT-13H39	AA	AA	AA	AA
F01	IFT-14H40	AA	AA	AA	AA
G01	IFT-15H48	GG	GG	GG	GG
H01	IFT-17H45	GG	GG	GG	GG
A02	IFT-18H47	GG	GG	GG	GG
B02	IFT-19H10	AA	AA	AA	AA
C02	ITT-21ET01	AG	AG	AG	AG
D02	ITT-22DT01	AA	AA	AA	AA
E02	ITT-23BT	GG	GG	GG	GG
F02	ITT-24J3	AA	AA	AA	AA
G02	ITT-25N2	GG	GG	GG	GG
H02	ITT-26K5	AA	AA	AA	AA
A03	BGBGN09D	AG	AG	AG	AG
B03	BGBGN12D	GG	GG	GG	GG
C03	BGBGN36D	GG	GG	GG	GG
D03	BGBGN31D	GG	GG	GG	GG
E03	BGBGN21D	AA	AA	AA	AA
F03	BGBGN20D	AA	AA	AA	AA
G03	BGBGN18D	AA	AA	AA	AA
A04	ITT-INDO-09	AA	AA	AA	AA
B04	ITT-INDO-10	AG	AG	AG	AG
C04	ITT-INDO-12	AA	AA	AA	AA
D04	ITT-INDO-14	AA	AA	AA	AA
E04	ITT-INDO-19	AG	AG	AG	AG
F04	ITT-INDO-20	AG	AG	AG	AG
G04	ITT-INDO-21	AG	AG	AG	AG
H04	ITT-INDO-30	AA	AA	AA	AA
A05	ITT-INDO-32	AG	AG	AG	AG
B05	ITT-INDO-33	AG	AG	AG	AG
C05	ITT-INDO-34	AG	AG	AG	AG
D05	ITT-INDO-37	AG	AG	AG	AG
E05	ITT-INDO-38	GG	GG	GG	GG
F05	ITT-INDO-40	AG	AG	AG	AG
G05	ITT-INDO-42	AG	AG	AG	AG
H05	ITT-INDO-43	AG	AG	AG	AG
A06	FBVN-25	GG	GG	GG	GG
B06	FBVN-26	GG	GG	GG	GG
C06	FBVN-27	GG	GG	GG	GG
D06	FBVN-28	GG	Not Determined	GG	GG
E06	FBVN-29	AG	AG	AG	AG
F06	FBVN-30	AG	AG	AG	AG
G06	FBVN-31	AG	AG	AG	AG
H06	FBVN-32	AG	AG	AG	AG

### *Application of KASP assay for high throughput Fec*^*B*^ *screening in global sheep breeds*

The KASP genotyping assay was further utilized to conduct largescale screening of global sheep breeds for the presence of Fec^B^ allele. A total of 1471 sheep representing 47 breeds from Africa, Europe, Latin America, South Asia, Southeast Asia, and West Asia were genotyped to establish the patterns of polymorphism across diverse genetic and geographic backgrounds. The investigated sheep samples from Africa (Burkina Faso, Ethiopia, Sudan), Europe (Austria, Bulgaria, Germany), Latin America (Argentina, Peru), and West Asia (Iran, Iraq) were completely monomorphic for the wild-type allele (A), with 100% of individuals genotyped as AA (Table 1). These Fec^B^ negative populations included indigenous and well-established breeds such as Djallonke, Mossi, Menz, Krainer Steinschaf, Corriedale, and Awassi, although genotyping a larger sample size and additional breeds may be warranted for further confirmation In contrast, Fec^B^ positive populations were identified exclusively in South Asia and Southeast Asia, specifically among sheep from Bangladesh, India, and Indonesia, where the mutation displayed variable levels of polymorphism and in some cases, near fixation.

In Bangladesh, local sheep populations from different regions such as North, East and Central parts of the country and the Bangladeshi Garole sheep carried the Fec^B^ allele. The Bangladesh Central sheep were nearly fixed for the mutant allele, with 95.5% Fec^BB^ (GG) and a Fec^B^ (G) allele frequency of 0.955. The Bangladesh East sheep revealed the lowest frequency of Fec^B^ allele (G) (0.300) in the country with the genotypic distribution of 45%, 50% and 5% of AA, AG, and GG individuals respectively. The Bangladesh North population had a relatively higher frequency of mutant Fec^B^ allele (G) (0.529) with equal distribution of different genotypes (AA = 29.4%, AG = 35.3%, GG = 35.3%). The Bangladeshi Garole sheep exhibited a predominance of the GG genotype (66.7%), with an allele frequency of G = 0.810. The results suggest both historical and ongoing natural selection for high fecundity in Bangladeshi sheep, where prolificacy is economically favoured.

In Indian sheep breeds, Fec^B^ polymorphism was observed in both indigenous and crossbred populations. The Indian Garole sheep were fixed for the GG genotype while the Garole × Sandyno crossbreds and Nilgiri × Sandyno crossbreds exhibited heterozygous and polymorphic profiles, with AG genotypes being dominant (G allele frequencies of 0.500 and 0.955, respectively). The NARI composite population was also predominantly GG (90.9%), with a G allele frequency of 0.955. The Nilgiri breed was exclusively AG (100%), indicating a balanced heterozygous state (A = 0.5, G = 0.5). Conversely, several indigenous Indian breeds including Kilakarsal, Mecheri, Madras Red, Nellore, Pattanam, and Vembur were fixed for the AA genotype, confirming that the Fec^B^ mutation remains confined to select populations and synthetic lines developed through controlled introgression. In Indonesian sheep, both the Indonesian Fat Tailed (IFT) and Indonesian Thin Tailed (ITT) breeds showed notable variations. The IFT population was composed predominantly of GG genotypes (64.7%) along with 17.6% AG genotypes, resulting in a G allele frequency of 0.735. The ITT population had a different composition with 64.5% AA, 23.7% AG, and 11.8% GG genotypes, resulting in G allele frequency of 0.237 (Table 1).

Altogether, the high-throughput screening indicated the absence of Fec^B^ mutation in the investigated sheep samples from Africa, Europe, Latin America, and West Asia while its presence was observed in select populations of Bangladesh, India, and Indonesia. Among Fec^B^ positive populations, near complete fixation of G allele was observed in Indian Garole and Bangladesh Central sheep, while Fec^B^ carriers were relatively more frequent in Nilgiri, Sandyno, and crossbreds.

## Discussion

Mutations in a closely linked group of genes under the Transforming growth factor-β (TGFβ) super family have been established to affect ovulation rate and litter size in sheep. Three genes of TGFβ family, Bone morphogenetic protein 15 (BMP15), Bone morphogenetic protein receptor IB (BMP15RIB) and Growth differentiation factor 9 (GDF9) code for proteins that are essential growth factors and receptors involved in follicular development. Variations in the structure and expression of these factors/receptors have been reported to have a significant effect on ovulation rate and litter size in sheep [26,27]. Several QTNs affecting litter size in sheep have been reported: ***Fec***_***B***_ (BMP15RIB) mutations in Garole, Booroola Merino, Javanese Thin Tail, Hu, Han and Kendrapada sheep [10,28], ***Fec***_***X***_ (BMP15) mutations in Romney (Inverdale-FecX^I^ and Hanna-FecX^H^), Belclare (FecX^B^), Belclare and Cambridge (Galway-FecX^G^), Lacaune (FecX^L^), Rasa Aragonesa (FecX^R^), Grivette (FecX^Gr^), Olkuska (FecX^O^) sheep [1,29–32] and ***Fec***_***G***_ (GDF9) mutations in Belclare and Cambridge (FecG^H^), Icelandic (Thoka-FecG^T^), Santa Ines (Embrapa-FecG^E^) sheep [29,32]. Among these, Bone Morphogenetic protein receptor 1B (BMP15RIB; FecB) has been the first major gene widely attempted by researchers for marker assisted introgression to improve litter size in sheep across various production systems [1]. With the possibility of achieving significant increase in fecundity in a single generation, several crossbreeding experiments were conducted, the results of which showed varying levels of advantage on ovulation rate, litter size and ewe productivity. This essentially depended on three major factors: donor and recipient genetic background, production system type, potential maternal effect of recipient ewes. In general, Booroola Merino, Garole, Hu and small tailed Han sheep were the FecB donors and the recipients included both wool and meat sheep breeds [1,33–35]. Booroola Merino was mostly the donor of choice in industrialized sheep production systems while Garole, Hu and Small Tailed Han breeds were used in low input production systems. A typical Fec^B^ introgression program involved initial crossbreeding for the transfer of favourable allele from the donor breed to a recipient followed by multiple backcrossing and inter crossing for one or more generations. Backcrossing enables recovery of most of the recipient’s genetic background with the retention of at least one copy of the favourable allele while the subsequent inter crossing helps to fix the allele in the recipient population. Availability of marker information on favourable allele can help both the processes of backcrossing and intercrossing by improving the effectiveness of foreground selection of target gene and the background selection of recipient genome.

Efficient, accurate, and scalable genotyping methods are central to the successful introgression of Fec^B^ in sheep breeding programs. In the current study, a competitive allele-specific PCR (KASP) assay was developed, validated, and deployed for high-throughput detection of the Fec^B^ mutation in diverse sheep populations. Initial approaches to Fec^B^ genotyping relied on PCR-RFLP, particularly using a forced restriction site with AvaII enzyme digestion [6,17]. This method, while robust and relatively accurate, is labor-intensive and time-consuming, requiring a post-PCR digestion step followed by gel electrophoresis for allele discrimination. Although suitable for small to moderate sample sizes, PCR-RFLP is suboptimal for large-scale screening initiatives where throughput and cost efficiency are critical [36,37]. Furthermore, incomplete digestion by restriction enzymes can result in genotyping errors, and the necessity for specialized restriction enzymes contributes to increased costs [38]. For instance, the single discordant case (FBVN-5) observed in the present study exemplifies this issue, where misinterpretation of a heterozygous individual as homozygous GG underscores the risk of allelic dropout in restriction-based assays. The observed genotyping error rate of 0.63% for PCR-RFLP in this study, though seemingly low, becomes significant in large-scale genetic studies, especially those informing marker-assisted selection or breeding decisions.

Subsequent advancements such as the Amplification Refractory Mutation System-PCR (ARMS-PCR) offered an improvement by eliminating the restriction digestion step. ARMS-PCR, utilizing allele-specific primers, enabled direct discrimination between wild-type and mutant alleles in separate reactions [39]. While it reduced assay time and costs, ARMS-PCR still require separate reactions for each allele, effectively doubling the workload and increasing the risk of sample handling errors. To overcome these limitations, tetra-primer ARMS-PCR was developed, employing a single-tube multiplex format with two allele-specific and two outer primers to amplify both alleles simultaneously along with a control band. This method improved convenience and reduced costs further [13]. However, the complexity of primer design and the need for careful optimization to prevent primer-dimer formation and non-specific amplification remained challenging, particularly for less-experienced laboratories [40,41]. Real-time PCR-based methods, particularly the TaqMan SNP genotyping assays, brought a significant leap in Fec^B^ genotyping technology [15]. By utilizing fluorescently labelled probes specific for each allele, TaqMan assays provided highly accurate, and rapid genotyping. However, the major limitation of TaqMan assays has been their high costs associated with probe synthesis [42].

In comparison, the KASP assay developed and validated in this study demonstrated several advantages making it highly suitable for large-scale Fec^B^ screening. Firstly, the KASP assay requires only three primers (two allele-specific and one common reverse primer) and utilizes a FRET-based signal generation system, making it significantly more cost-effective while retaining the closed-tube, high-throughput advantage. Genotyping in the KASP assay is determined by differential fluorescence of two dyes (FAM and HEX) corresponding to each allele, enabling clear discrimination of homozygous and heterozygous genotypes. Validation of the KASP assay showed 100% concordance with sequencing results across 224 individuals representing diverse geographic and genetic backgrounds, underscoring the assay’s high specificity and sensitivity. Additionally, the assay exhibited high genotyping accuracy across multiple real-time PCR platforms (Bio-Rad CFX96, Roche LightCycler 480 II, ABI QuantStudio 6), confirming its versatility and cross-platform robustness. This cross-platform compatibility is particularly valuable for breeding programs in countries where diverse equipment infrastructures are available. Furthermore, the KASP assay’s ability to perform genotyping directly after a standard touch-down PCR without the need for post-PCR processing minimizes technical complexity and contamination risks, streamlining workflows for large-scale studies. The assay is also flexible in that the touchdown PCR can be performed using a standard thermal cycler, and the subsequent endpoint measurements can be done on a real-time PCR instrument or a fluorescence plate reader. This is a key advantage, as it provides a wide range of detection options and facilitates adaptation to different laboratory settings. The KASP method maintains equivalent sensitivity and accuracy similar to Taqman assays but at a significantly lower per-sample cost. This cost efficiency arises because KASP does not require expensive dual-labeled probes, relying instead on standard oligonucleotides and universal FRET cassettes [42]. This aspect is especially critical when genotyping thousands of animals in national-scale or regional Fec^B^ introgression or marker assisted selection programs in sheep. When compared to tetra-primer ARMS-PCR, the KASP assay offers improved ease of use and higher throughput capacity. Although both methods are cost-effective and avoid enzymatic digestion, the KASP assay employs fluorescence-based detection, enabling objective and automated allele discrimination, thereby eliminating the subjectivity associated with gel band interpretation. This greatly reduces the chances of human error and enhances the reproducibility of genotyping results across laboratories.

The large-scale genotyping across 1471 sheep from 47 breeds using KASP assay in this study further demonstrates its scalability and practical utility. Patterns of Fec^B^ distribution revealed absence of the mutation in the investigated African, European, Latin American, and West Asian populations, while polymorphism and even fixation were observed in several South and Southeast Asian breeds, particularly in Bangladesh, India, and Indonesia. Among Indonesian sheep, the Indonesian Fat-Tailed sheep (IFT) exhibited a moderate Fec^B^ (G) allele frequency of 0.735, while the Indonesian Thin-Tailed (ITT) sheep presented a considerably lower Fec^B^ (G) allele frequency of 0.237. These findings corroborate earlier reports indicating the presence of Fec^B^ polymorphism within Indonesian indigenous breeds, albeit at varying frequencies [8]. Importantly, the IFT population demonstrated a relatively high occurrence of the homozygous mutant genotype Fec^BB^ (GG, 64.7%), suggesting significant potential for utilizing these animals in selection programs targeting higher prolificacy. Indian sheep breeds exhibited diverse Fec^B^ allele distributions. The Garole breed, hypothesized as the origin of the Booroola mutation [6], was found to be fixed for the G allele in the present study. Other Indian breeds such as Nilgiri and NARI Composite showed moderate frequencies of the G allele (0.5 and 0.955, respectively), supporting earlier findings [43,44]. In contrast, the Mecheri, Madras Red, Kilakarsal, and Pattanam breeds displayed absence of the Fec^B^ mutation, remaining entirely wild type at the tested locus. This variability reflects the historical segregation of Fec^B^ variations and recent attempts of introgression into breeds/populations for improved prolificacy. Recent reports [45] corroborate these findings on the presence of Fec^B^ in Nilgiri and Sandyno breeds in India and the introgression success in NARI composites within Tamil Nadu [46].

In Bangladesh, Garole sheep showed a high Fec^B^ allele frequency (0.810), comparable to their Indian counterparts. However, other regional populations exhibited heterogeneous distributions: Bangladesh East (0.300), Bangladesh North (0.529), and Bangladesh Central (0.955). Despite this heterogeneity, the present study reveals the distribution of Fec^B^ mutation is relatively high across local sheep populations in Bangladesh. This is comparable to FecB variations in Garole sheep of India and certain prolific Chinese sheep breeds. Hu sheep and Small Tail Han (STH) sheep populations have been shown to carry high frequencies of the Fec^B^ allele, contributing significantly to their prolificacy profiles [34,47]. For instance, the Hu breed was reported to be almost entirely homozygous for the Fec^B^ mutation (BB genotype) while Chinese Merino prolific meat strains presented notable frequency of mutant allele reinforcing its role in enhanced reproductive performance. A recent study [48] highlighted BMPR1B polymorphisms in other Chinese sheep as well with a moderate frequency of Fec^B^ (G allele) in Wadi (0.59) and low frequency in Tan sheep (0.07). However, in the East Asian context beyond China, Japanese and Mongolian sheep populations generally lack the Fec^B^ mutation, indicating the mutation has not spread uniformly across the entire East Asian region [34].

An integrative analysis of the allele frequencies reveals that high Fec^B^ (G) allele frequencies were observed in Garole sheep from India and Bangladesh, Hu and Small Tail Han sheep from China, and Indonesian Fat-Tailed sheep. These findings emphasize that the Fec^B^ mutation, while geographically dispersed, remains relatively restricted to specific high-prolificacy genetic lineages and is rare or absent in sheep adapted to arid and semi-arid environments. It is noteworthy that in populations where the Fec^B^ mutation is fixed or highly prevalent, there is a corresponding improvement in litter size traits, often with a significant additive effect. Homozygous carriers (BB) generally produced approximately 1.5 lambs more per lambing than non-carriers, while heterozygous carriers (B+) showed intermediate increases in litter size [8,34,47]. Nevertheless, while the Fec^B^ mutation offers substantial gains in fecundity, it must be emphasized that its introduction into low-prolificacy breeds must be carefully managed to avoid potential drawbacks such as reduced lamb birth weight, increased perinatal mortality, and challenges in maternal rearing capacity [1,49]. Such trade-offs underscore the necessity for comprehensive breeding strategies that balance reproductive performance with lamb survival and ewe fitness.

One of the most notable findings of the present study is the relatively high frequency of Fec^B^ mutations observed in native sheep populations across various regions of Bangladesh. This presents a unique opportunity to harness the prolificacy potential for improving sheep productivity and profitability among smallholder farmers. Exploiting the Fec^B^ mutation in Bangladesh could substantially enhance lambing rates, leading to increased numbers of lambs per ewe per year. Such an increase can translate into higher flock turnover and greater meat production, thus enhancing the income for smallholders operating under low-input systems. Marker-assisted selection (MAS) strategies can be employed to selectively propagate animals carrying Fec^B^ allele, particularly the heterozygous (B+) and homozygous mutant (BB) genotypes, thereby accelerating genetic gain for prolificacy traits in village sheep flocks. Furthermore, controlled breeding programs could utilize homozygous Fec^BB^ rams to maximize the transmission of mutant allele across wider sheep populations. However, it is crucial that selection for prolificacy be complemented by improvements in management practices, particularly nutrition and lamb rearing support, to mitigate potential challenges associated with multiple births, such as lower birth weights and increased neonatal mortality. Establishing nucleus breeding flocks in regional centers, coupled with systematic dissemination of Fec^B^ positive breeding rams to farmers, could create a sustainable genetic improvement program tailored for Bangladeshi conditions. Ultimately, leveraging the naturally high prevalence of Fec^B^ mutation in Bangladeshi sheep offers a scientifically sound, economically viable pathway for enhancing smallholder profitability and contributing to rural livelihoods through increased and more consistent sheep meat production.

## Conclusion

The present study reports the development and validation of a simple and cost-effective competitive allele-specific PCR (KASP) assay for the detection of Fec^B^ mutation in sheep. The assay showed complete concordance with sequencing data across diverse breeds and demonstrated consistent performance with reliability and specificity across multiple real-time PCR platforms. Application of the assay to screening of a large number of sheep breeds from diverse geographical location revealed the Fec^B^ mutation is largely confined to specific South and Southeast Asian sheep populations, notably in Bangladesh, India, and Indonesia, while it was not observed in the breeds investigated from Africa, Europe, Latin America, and West Asia. High frequencies of the mutant allele were observed in select populations such as the Garole, Bangladesh Central, and NARI Composite breeds, reflecting both historical segregation and recent introgression efforts aimed at enhancing prolificacy. The KASP assay, by virtue of its low cost, scalability, and ease of implementation, represents a significant advancement over existing genotyping methodologies such as PCR-RFLP and TaqMan assays. It provides an effective tool for large-scale marker-assisted selection and introgression programs targeting reproductive traits in sheep, particularly in resource-constrained settings. Future breeding programs should, however, carefully integrate selection for prolificacy with comprehensive management practices to optimize both reproductive efficiency and lamb survival outcomes.

## Supporting information

S1 File Declaration on Inclusivity in Global Research. (DOCX)

S2 File Validation of competitive allele specific PCR genotypes of BMPR1B locus using PCR-RFLP and targeted sequencing approaches. (DOCX)

S3 File PCR-RFLP genotyping of BMPR1B locus using restriction enzyme Ava II. (Size of undigested PCR products −190 bp; After restriction digestion, AA genotypes showed a single band of 190 bp; GG genotypes showed a single band of 160 bp; AG genotypes showed double bands of 190 and 160 bp; A 50 bp standard size marker ladder was used for resolving band size).
(DOCX)

S4 File Raw images of PCR-RFLP genotyping of BMPR1B locus using restriction enzyme Ava II. (PDF)
