# Supplementary material for: Development and validation of a simple, cost-effective competitive allele-specific PCR assay for largescale screening and detection of FecB mutation in sheep
Source: PLoS One. 2025 Dec 2;20(12):e0337392. doi: 10.1371/journal.pone.0337392 (PMC12671827; doi:10.1371/journal.pone.0337392)
Supplement: S2 File — (DOCX) [file pone.0337392.s002.docx]

S1 File. Validation of competitive allele specific PCR genotypes of BMPR1B locus using PCR-RFLP and targeted sequencing approaches

| Country | Breed | Breed code | Sample Name | BioRad CFX96 Genotype | PCR-RFLP | Sequence | NCBI Acc.No. |
| --- | --- | --- | --- | --- | --- | --- | --- |
| Austria | KrainerSteinschaf | KSF | KS1 | AA | - | AA | OR767505 |
| Austria | KrainerSteinschaf | KSF | KS12 | AA | - | AA | OR767506 |
| Austria | KrainerSteinschaf | KSF | KS3 | AA | - | AA | OR767507 |
| Austria | KrainerSteinschaf | KSF | SH-AT-KS-34 | AA | AA | AA | OR767644 |
| Austria | KrainerSteinschaf | KSF | SH-AT-KS-35 | AA | AA | AA | OR767645 |
| Austria | KrainerSteinschaf | KSF | SH-AT-KS-8 | AA | AA | AA | OR767646 |
| Austria | Mouflon | MUF | MUF1 | AA | AA | AA | OR767508 |
| Austria | Texel | TEX | TEX3 | AA | AA | AA | OR767511 |
| Bangladesh | Bangladesh Central | BGC | BGBGC04D | GG | GG | GG | OR767429 |
| Bangladesh | Bangladesh Central | BGC | BGBGC12D | AA | AA | AA | OR767430 |
| Bangladesh | Bangladesh Central | BGC | BGBGC13D | GG | GG | GG | OR767431 |
| Bangladesh | Bangladesh Central | BGC | BGBGC14D | GG | GG | GG | OR767432 |
| Bangladesh | Bangladesh Central | BGC | BGBGC18D | GG | GG | GG | OR767433 |
| Bangladesh | Bangladesh Central | BGC | BGBGC20D | GG | GG | GG | OR767434 |
| Bangladesh | Bangladesh Central | BGC | BGBGC22D | GG | GG | GG | OR767435 |
| Bangladesh | Bangladesh Central | BGC | BGBGC23D | GG | GG | GG | OR767436 |
| Bangladesh | Bangladesh Central | BGC | BGBGC38D | GG | GG | GG | OR767437 |
| Bangladesh | Bangladesh Central | BGC | BGBGC39D | GG | GG | GG | OR767438 |
| Bangladesh | Bangladesh Central | BGC | BGC-02 | GG | GG | GG | OR767446 |
| Bangladesh | Bangladesh Central | BGC | BGC-03 | GG | GG | GG | OR767447 |
| Bangladesh | Bangladesh Central | BGC | BGC-05 | GG | GG | GG | OR767448 |
| Bangladesh | Bangladesh Central | BGC | BGC-08 | GG | GG | GG | OR767449 |
| Bangladesh | Bangladesh Central | BGC | BGC-10 | GG | GG | GG | OR767450 |
| Bangladesh | Bangladesh Central | BGC | BGC-11 | GG | GG | GG | OR767451 |
| Bangladesh | Bangladesh Central | BGC | BGC-15 | GG | GG | GG | OR767452 |
| Bangladesh | Bangladesh Central | BGC | BGC-16 | GG | GG | GG | OR767453 |
| Bangladesh | Bangladesh Central | BGC | BGC-19 | GG | GG | GG | OR767454 |
| Bangladesh | Bangladesh Central | BGC | BGC-21 | GG | GG | GG | OR767455 |
| Bangladesh | Bangladesh Central | BGC | BGC-26 | GG | GG | GG | OR767456 |
| Bangladesh | Bangladesh Central | BGC | BGC-37 | GG | GG | GG | OR767457 |
| Bangladesh | Bangladesh East | BGE | BGE-01 | AA | AA | AA | OR767459 |
| Bangladesh | Bangladesh East | BGE | BGE-05 | AA | AA | AA | OR767460 |
| Bangladesh | Bangladesh East | BGE | BGE-17 | AG | AG | AG | OR767461 |
| Bangladesh | Bangladesh East | BGE | BGE-19 | AG | AG | AG | OR767462 |
| Bangladesh | Bangladesh East | BGE | BGE-20 | AA | AA | AA | OR767463 |
| Bangladesh | Bangladesh East | BGE | BGE-22 | AG | AG | AG | OR767464 |
| Bangladesh | Bangladesh East | BGE | BGE-23 | AG | AG | AG | OR767465 |
| Bangladesh | Bangladesh East | BGE | BGE-25 | AG | AG | AG | OR767466 |
| Bangladesh | Bangladesh East | BGE | BGE-26 | AG | AG | AG | OR767467 |
| Bangladesh | Bangladesh East | BGE | BGE-27 | AA | AA | AA | OR767468 |
| Bangladesh | Bangladesh East | BGE | BGE-28 | AG | AG | AG | OR767469 |
| Bangladesh | Bangladesh East | BGE | BGE-29 | AG | AG | AG | OR767470 |
| Bangladesh | Bangladesh East | BGE | BGE-30 | GG | GG | GG | OR767471 |
| Bangladesh | Bangladesh East | BGE | BGE-31 | AG | AG | AG | OR767472 |
| Bangladesh | Bangladesh East | BGE | BGE-33 | AA | AA | AA | OR767473 |
| Bangladesh | Bangladesh East | BGE | BGE-37 | AA | AA | AA | OR767474 |
| Bangladesh | Bangladesh East | BGE | BGE-38 | AA | AA | AA | OR767475 |
| Bangladesh | Bangladesh East | BGE | BGE-40 | AA | AA | AA | OR767476 |
| Bangladesh | Bangladesh North | BGN | BGBGN09D | AG | - | AG | OR767439 |
| Bangladesh | Bangladesh North | BGN | BGBGN12D | GG | - | GG | OR767440 |
| Bangladesh | Bangladesh North | BGN | BGBGN18D | AA | - | AA | OR767441 |
| Bangladesh | Bangladesh North | BGN | BGBGN20D | AA | - | AA | OR767442 |
| Bangladesh | Bangladesh North | BGN | BGBGN21D | AA | - | AA | OR767443 |
| Bangladesh | Bangladesh North | BGN | BGBGN31D | GG | - | GG | OR767444 |
| Bangladesh | Bangladesh North | BGN | BGBGN36D | GG | - | GG | OR767445 |
| Bangladesh | Bangladesh North | BGN | BGD-BGN11 | AG | - | AG | OR767458 |
| Bangladesh | Bangladesh North | BGN | BGN-01 | AG | AG | AG | OR767477 |
| Bangladesh | Bangladesh North | BGN | BGN-03 | GG | GG | GG | OR767478 |
| Bangladesh | Bangladesh North | BGN | BGN-04 | AG | AG | AG | OR767479 |
| Bangladesh | Bangladesh North | BGN | BGN-19 | AG | AG | AG | OR767480 |
| Bangladesh | Garole | GAR | GAR-09 | AG | AG | AG | OR767482 |
| Bangladesh | Garole | GAR | GAR-11 | GG | GG | GG | OR767483 |
| Bangladesh | Garole | GAR | GAR-13 | GG | GG | GG | OR767484 |
| Bangladesh | Garole | GAR | GAR-14D | GG | GG | GG | OR767485 |
| Bangladesh | Garole | GAR | GAR-17D | GG | GG | GG | OR767486 |
| Bangladesh | Garole | GAR | GAR-18D | GG | GG | GG | OR767487 |
| Bangladesh | Garole | GAR | GAR-19D | AG | AG | AG | OR767488 |
| Bangladesh | Garole | GAR | GAR-20D | GG | GG | GG | OR767489 |
| Bangladesh | Garole | GAR | GAR-21D | GG | GG | GG | OR767490 |
| Bangladesh | Garole | GAR | GAR-22D | GG | GG | GG | OR767491 |
| Bangladesh | Garole | GAR | GAR-23D | AG | AG | AG | OR767492 |
| Bangladesh | Garole | GAR | GAR-24D | GG | GG | GG | OR767493 |
| Bangladesh | Garole | GAR | GAR-29D | GG | GG | GG | OR767494 |
| Bangladesh | Garole | GAR | GAR-30D | AG | AG | AG | OR767495 |
| Bangladesh | Garole | GAR | GAR-31D | AA | AA | AA | OR767496 |
| Bangladesh | Garole | GAR | GAR-32D | GG | GG | GG | OR767497 |
| Bangladesh | Garole | GAR | GAR-34D | AG | AG | AG | OR767498 |
| Bangladesh | Garole | GAR | GAR-35D | GG | GG | GG | OR767499 |
| Bangladesh | Garole | GAR | GAR-37D | GG | GG | GG | OR767500 |
| Bangladesh | Garole | GAR | GAR-39D | GG | GG | GG | OR767501 |
| Bulgaria | Karakachanska | KAR | KAR13 | AA | AA | AA | OR767504 |
| Bulgaria | Shumenska | SHM | SHM11 | AA | AA | AA | OR767510 |
| Burkina Faso | Djallonke | DJA | BF-573 | AA | AA | AA | OR767427 |
| Burkina Faso | Djallonke | DJA | BF-574 | AA | AA | AA | OR767428 |
| Germany | Bergschaf | BER | BERG19 | AA | AA | AA | OR767481 |
| INDIA | GaroleXSandyno cross | GAS | FBVN-44 | AG | AG | AG | OR767555 |
| INDIA | GaroleXSandyno cross | GAS | FBVN-45 | AG | AG | AG | OR767556 |
| INDIA | GaroleXSandyno cross | GAS | FBVN-46 | AG | AG | AG | OR767557 |
| INDIA | GaroleXSandyno cross | GAS | FBVN-47 | AG | AG | AG | OR767558 |
| INDIA | GaroleXSandyno cross | GAS | FBVN-48 | AG | AG | AG | OR767559 |
| INDIA | Garole | IGR | FBVN-12 | GG | GG | GG | OR767523 |
| INDIA | Garole | IGR | FBVN-13 | GG | GG | GG | OR767524 |
| INDIA | Garole | IGR | FBVN-14 | GG | GG | GG | OR767525 |
| INDIA | Garole | IGR | FBVN-15 | GG | GG | GG | OR767526 |
| INDIA | Garole | IGR | FBVN-16 | GG | GG | GG | OR767527 |
| INDIA | Garole | IGR | FBVN-17 | GG | GG | GG | OR767528 |
| INDIA | Garole | IGR | FBVN-18 | GG | GG | GG | OR767529 |
| INDIA | NARI Composite | NAR | FBVN-1 | GG | GG | GG | OR767512 |
| INDIA | NARI Composite | NAR | FBVN-2 | GG | GG | GG | OR767513 |
| INDIA | NARI Composite | NAR | FBVN-3 | GG | GG | GG | OR767514 |
| INDIA | NARI Composite | NAR | FBVN-4 | GG | GG | GG | OR767515 |
| **INDIA** | **NARI Composite** | **NAR** | **FBVN-5** | **AG** | **GG** | **AG** | **OR767516** |
| INDIA | NARI Composite | NAR | FBVN-6 | GG | GG | GG | OR767517 |
| INDIA | NARI Composite | NAR | FBVN-7 | GG | GG | GG | OR767518 |
| INDIA | NARI Composite | NAR | FBVN-8 | GG | GG | GG | OR767519 |
| INDIA | NARI Composite | NAR | FBVN-9 | GG | GG | GG | OR767520 |
| INDIA | NARI Composite | NAR | FBVN-10 | GG | GG | GG | OR767521 |
| INDIA | NARI Composite | NAR | FBVN-11 | GG | GG | GG | OR767522 |
| INDIA | Nilgiri | NIL | FBVN-29 | AG | AG | AG | OR767540 |
| INDIA | Nilgiri | NIL | FBVN-30 | AG | AG | AG | OR767541 |
| INDIA | Nilgiri | NIL | FBVN-31 | AG | AG | AG | OR767542 |
| INDIA | Nilgiri | NIL | FBVN-32 | AG | AG | AG | OR767543 |
| INDIA | Nilgiri | NIL | FBVN-33 | AG | AG | AG | OR767544 |
| INDIA | Nilgiri | NIL | FBVN-34 | AG | AG | AG | OR767545 |
| INDIA | Nilgiri | NIL | FBVN-35 | AG | AG | AG | OR767546 |
| INDIA | Nilgiri | NIL | FBVN-36 | AG | AG | AG | OR767547 |
| INDIA | NilgiriXSandyno cross | NIS | FBVN-19 | GG | GG | GG | OR767530 |
| INDIA | NilgiriXSandyno cross | NIS | FBVN-20 | GG | GG | GG | OR767531 |
| INDIA | NilgiriXSandyno cross | NIS | FBVN-21 | GG | GG | GG | OR767532 |
| INDIA | NilgiriXSandyno cross | NIS | FBVN-22 | GG | GG | GG | OR767533 |
| INDIA | NilgiriXSandyno cross | NIS | FBVN-23 | GG | GG | GG | OR767534 |
| INDIA | NilgiriXSandyno cross | NIS | FBVN-24 | GG | GG | GG | OR767535 |
| INDIA | NilgiriXSandyno cross | NIS | FBVN-25 | GG | GG | GG | OR767536 |
| INDIA | NilgiriXSandyno cross | NIS | FBVN-26 | GG | GG | GG | OR767537 |
| INDIA | NilgiriXSandyno cross | NIS | FBVN-27 | GG | GG | GG | OR767538 |
| INDIA | NilgiriXSandyno cross | NIS | FBVN-28 | GG | GG | GG | OR767539 |
| INDIA | NilgiriXSandyno cross | NIS | FBVN-41 | AG | AG | AG | OR767552 |
| INDIA | Sandyno | SAN | FBVN-37 | AG | AG | AG | OR767548 |
| INDIA | Sandyno | SAN | FBVN-38 | AA | - | AA | OR767549 |
| INDIA | Sandyno | SAN | FBVN-39 | AG | AG | AG | OR767550 |
| INDIA | Sandyno | SAN | FBVN-40 | AG | AG | AG | OR767551 |
| INDIA | Sandyno | SAN | FBVN-42 | AG | AG | AG | OR767553 |
| INDIA | Sandyno | SAN | FBVN-43 | AA | - | AA | OR767554 |
| Indonesia | Indonesian Fat Tailed | IFT | IFT-10N03 | AG | - | AG | OR767563 |
| Indonesia | Indonesian Fat Tailed | IFT | IFT-11H4 | AG | - | AG | OR767564 |
| Indonesia | Indonesian Fat Tailed | IFT | IFT-12N04 | GG | - | GG | OR767565 |
| Indonesia | Indonesian Fat Tailed | IFT | IFT-13H39 | AA | - | AA | OR767566 |
| Indonesia | Indonesian Fat Tailed | IFT | IFT-14H40 | AA | - | AA | OR767567 |
| Indonesia | Indonesian Fat Tailed | IFT | IFT-15H48 | GG | - | GG | OR767568 |
| Indonesia | Indonesian Fat Tailed | IFT | IFT-17H45 | GG | - | GG | OR767569 |
| Indonesia | Indonesian Fat Tailed | IFT | IFT-18H47 | GG | - | GG | OR767570 |
| Indonesia | Indonesian Fat Tailed | IFT | IFT-19H10 | AA | - | AA | OR767571 |
| Indonesia | Indonesian Fat Tailed | IFT | IFT-1N01 | AG | - | AG | OR767572 |
| Indonesia | Indonesian Fat Tailed | IFT | IFT-2H12 | GG | - | GG | OR767573 |
| Indonesia | Indonesian Fat Tailed | IFT | IFT-3H32 | GG | - | GG | OR767574 |
| Indonesia | Indonesian Fat Tailed | IFT | IFT-5H49 | GG | - | GG | OR767575 |
| Indonesia | Indonesian Fat Tailed | IFT | IFT-6H43 | GG | - | GG | OR767576 |
| Indonesia | Indonesian Fat Tailed | IFT | IFT-7H23 | GG | - | GG | OR767577 |
| Indonesia | Indonesian Fat Tailed | IFT | IFT-8H5 | GG | - | GG | OR767578 |
| Indonesia | Indonesian Fat Tailed | IFT | IFT-9H2 | GG | - | GG | OR767579 |
| Indonesia | Indonesian Thin Tailed | ITT | INDO-21 | AG | - | AG | OR767647 |
| Indonesia | Indonesian Thin Tailed | ITT | INDO2-106 | AA | AA | AA | OR767580 |
| Indonesia | Indonesian Thin Tailed | ITT | INDO2-107 | AA | AA | AA | OR767581 |
| Indonesia | Indonesian Thin Tailed | ITT | INDO2-108 | AA | AA | AA | OR767582 |
| Indonesia | Indonesian Thin Tailed | ITT | INDO2-111 | AA | AA | AA | OR767583 |
| Indonesia | Indonesian Thin Tailed | ITT | INDO2-114 | AA | AA | AA | OR767584 |
| Indonesia | Indonesian Thin Tailed | ITT | INDO2-116 | AA | AA | AA | OR767585 |
| Indonesia | Indonesian Thin Tailed | ITT | INDO2-117 | AA | AA | AA | OR767586 |
| Indonesia | Indonesian Thin Tailed | ITT | INDO2-118 | AA | AA | AA | OR767587 |
| Indonesia | Indonesian Thin Tailed | ITT | INDO2-122 | AA | AA | AA | OR767588 |
| Indonesia | Indonesian Thin Tailed | ITT | INDO2-127 | AA | AA | AA | OR767589 |
| Indonesia | Indonesian Thin Tailed | ITT | INDO2-132 | AA | AA | AA | OR767590 |
| Indonesia | Indonesian Thin Tailed | ITT | INDO2-15 | AA | AA | AA | OR767591 |
| Indonesia | Indonesian Thin Tailed | ITT | INDO2-25 | AA | AA | AA | OR767592 |
| Indonesia | Indonesian Thin Tailed | ITT | INDO2-26 | AG | AG | AG | OR767593 |
| Indonesia | Indonesian Thin Tailed | ITT | INDO2-62 | GG | GG | GG | OR767594 |
| Indonesia | Indonesian Thin Tailed | ITT | INDO2-70 | AA | AA | AA | OR767595 |
| Indonesia | Indonesian Thin Tailed | ITT | INDO2-75 | AG | AG | AG | OR767596 |
| Indonesia | Indonesian Thin Tailed | ITT | INDO2-87 | AA | AA | AA | OR767597 |
| Indonesia | Indonesian Thin Tailed | ITT | INDO2-98 | AA | AA | AA | OR767598 |
| Indonesia | Indonesian Thin Tailed | ITT | INDO-30 | AA | - | AA | OR767649 |
| Indonesia | Indonesian Thin Tailed | ITT | INDO-42 | AG | - | AG | OR767650 |
| Indonesia | Indonesian Thin Tailed | ITT | INDO-43 | AG | - | AG | OR767648 |
| Indonesia | Indonesian Thin Tailed | ITT | ITT-21ET01 | AG | - | AG | OR767599 |
| Indonesia | Indonesian Thin Tailed | ITT | ITT-22DT01 | AA | - | AA | OR767600 |
| Indonesia | Indonesian Thin Tailed | ITT | ITT-23BT | GG | - | GG | OR767601 |
| Indonesia | Indonesian Thin Tailed | ITT | ITT-24J3 | AA | - | AA | OR767602 |
| Indonesia | Indonesian Thin Tailed | ITT | ITT-25N2 | GG | - | GG | OR767603 |
| Indonesia | Indonesian Thin Tailed | ITT | ITT-26K5 | AA | - | AA | OR767604 |
| Indonesia | Indonesian Thin Tailed | ITT | ITT-27I3 | AA | - | AA | OR767605 |
| Indonesia | Indonesian Thin Tailed | ITT | ITT-29CT9 | GG | - | GG | OR767606 |
| Indonesia | Indonesian Thin Tailed | ITT | ITT-30CT8 | AA | - | AA | OR767607 |
| Indonesia | Indonesian Thin Tailed | ITT | ITT-31CT7 | AA | - | AA | OR767608 |
| Indonesia | Indonesian Thin Tailed | ITT | ITT-33CT2 | AA | - | AA | OR767609 |
| Indonesia | Indonesian Thin Tailed | ITT | ITT-35ET02 | AG | - | AG | OR767610 |
| Indonesia | Indonesian Thin Tailed | ITT | ITT-36ET04 | GG | - | GG | OR767611 |
| Indonesia | Indonesian Thin Tailed | ITT | ITT-37CT3 | AG | - | AG | OR767612 |
| Indonesia | Indonesian Thin Tailed | ITT | ITT-38CT6 | GG | - | GG | OR767613 |
| Indonesia | Indonesian Thin Tailed | ITT | ITT-39CT4 | AG | - | AG | OR767614 |
| Indonesia | Indonesian Thin Tailed | ITT | ITT-40CT5 | AA | - | AA | OR767615 |
| Indonesia | Indonesian Thin Tailed | ITT | ITT-INDO-09 | AA | - | AA | OR767616 |
| Indonesia | Indonesian Thin Tailed | ITT | ITT-INDO-10 | AG | - | AG | OR767619 |
| Indonesia | Indonesian Thin Tailed | ITT | ITT-INDO-101 | AA | AA | AA | OR767617 |
| Indonesia | Indonesian Thin Tailed | ITT | ITT-INDO-103 | AA | AA | AA | OR767618 |
| Indonesia | Indonesian Thin Tailed | ITT | ITT-INDO-12 | AA | - | AA | OR767622 |
| Indonesia | Indonesian Thin Tailed | ITT | ITT-INDO-121 | AA | AA | AA | OR767620 |
| Indonesia | Indonesian Thin Tailed | ITT | ITT-INDO-124 | AA | AA | AA | OR767621 |
| Indonesia | Indonesian Thin Tailed | ITT | ITT-INDO-14 | AA | - | AA | OR767623 |
| Indonesia | Indonesian Thin Tailed | ITT | ITT-INDO-19 | AG | - | AG | OR767624 |
| Indonesia | Indonesian Thin Tailed | ITT | ITT-INDO-20 | AG | - | AG | OR767625 |
| Indonesia | Indonesian Thin Tailed | ITT | ITT-INDO-32 | AG | - | AG | OR767626 |
| Indonesia | Indonesian Thin Tailed | ITT | ITT-INDO-33 | AG | - | AG | OR767627 |
| Indonesia | Indonesian Thin Tailed | ITT | ITT-INDO-34 | AG | - | AG | OR767628 |
| Indonesia | Indonesian Thin Tailed | ITT | ITT-INDO-37 | AG | - | AG | OR767629 |
| Indonesia | Indonesian Thin Tailed | ITT | ITT-INDO-38 | GG | - | GG | OR767630 |
| Indonesia | Indonesian Thin Tailed | ITT | ITT-INDO-40 | AG | - | AG | OR767631 |
| Indonesia | Indonesian Thin Tailed | ITT | ITT-INDO-44 | AG | AG | AG | OR767632 |
| Indonesia | Indonesian Thin Tailed | ITT | ITT-INDO-46 | GG | GG | GG | OR767633 |
| Indonesia | Indonesian Thin Tailed | ITT | ITT-INDO-47 | AA | AA | AA | OR767634 |
| Indonesia | Indonesian Thin Tailed | ITT | ITT-INDO-50 | AA | AA | AA | OR767635 |
| Indonesia | Indonesian Thin Tailed | ITT | ITT-INDO-51 | AG | AG | AG | OR767636 |
| Indonesia | Indonesian Thin Tailed | ITT | ITT-INDO-53 | AG | AG | AG | OR767637 |
| Indonesia | Indonesian Thin Tailed | ITT | ITT-INDO-69 | GG | GG | GG | OR767638 |
| Indonesia | Indonesian Thin Tailed | ITT | ITT-INDO-73 | AG | AG | AG | OR767639 |
| Indonesia | Indonesian Thin Tailed | ITT | ITT-INDO-93 | AA | AA | AA | OR767640 |
| Indonesia | Indonesian Thin Tailed | ITT | ITT-INDO-95 | AA | AA | AA | OR767641 |
| Iran | Shal | SHA | SHA18 | AA | AA | AA | OR767509 |
| Iraq | Hamdani | HAM | HAM30 | AA | AA | AA | OR767560 |
| Iraq | Hamdani | HAM | HAM6 | AA | AA | AA | OR767502 |
| Iraq | Hamdani | HAM | HAM60 | AA | AA | AA | OR767561 |
| Iraq | Hamdani | HAM | HAM834 | AA | AA | AA | OR767562 |
| Peru | Junin | JNN | JNN33 | AA | - | AA | OR767642 |
| Peru | Junin | JNN | JNN70 | AA | - | AA | OR767643 |
| Peru | Junin | JNN | JUNIN17 | AA | AA | AA | OR767503 |
