# Supplementary material for: Development and validation of a simple, cost-effective competitive allele-specific PCR assay for largescale screening and detection of FecB mutation in sheep
Source: PLoS One. 2025 Dec 2;20(12):e0337392. doi: 10.1371/journal.pone.0337392 (PMC12671827; doi:10.1371/journal.pone.0337392)

S2 File. PCR-RFLP genotyping of BMPR1B locus using restriction enzyme Ava II. (Size of undigested PCR products -190bp; After restriction digestion, AA genotypes showed a single band of 190 bp; GG genotypes showed a single band of 160bp; AG genotypes showed double bands of 190 and 160bp; A 50 bp standard size marker ladder was used for resolving band size)


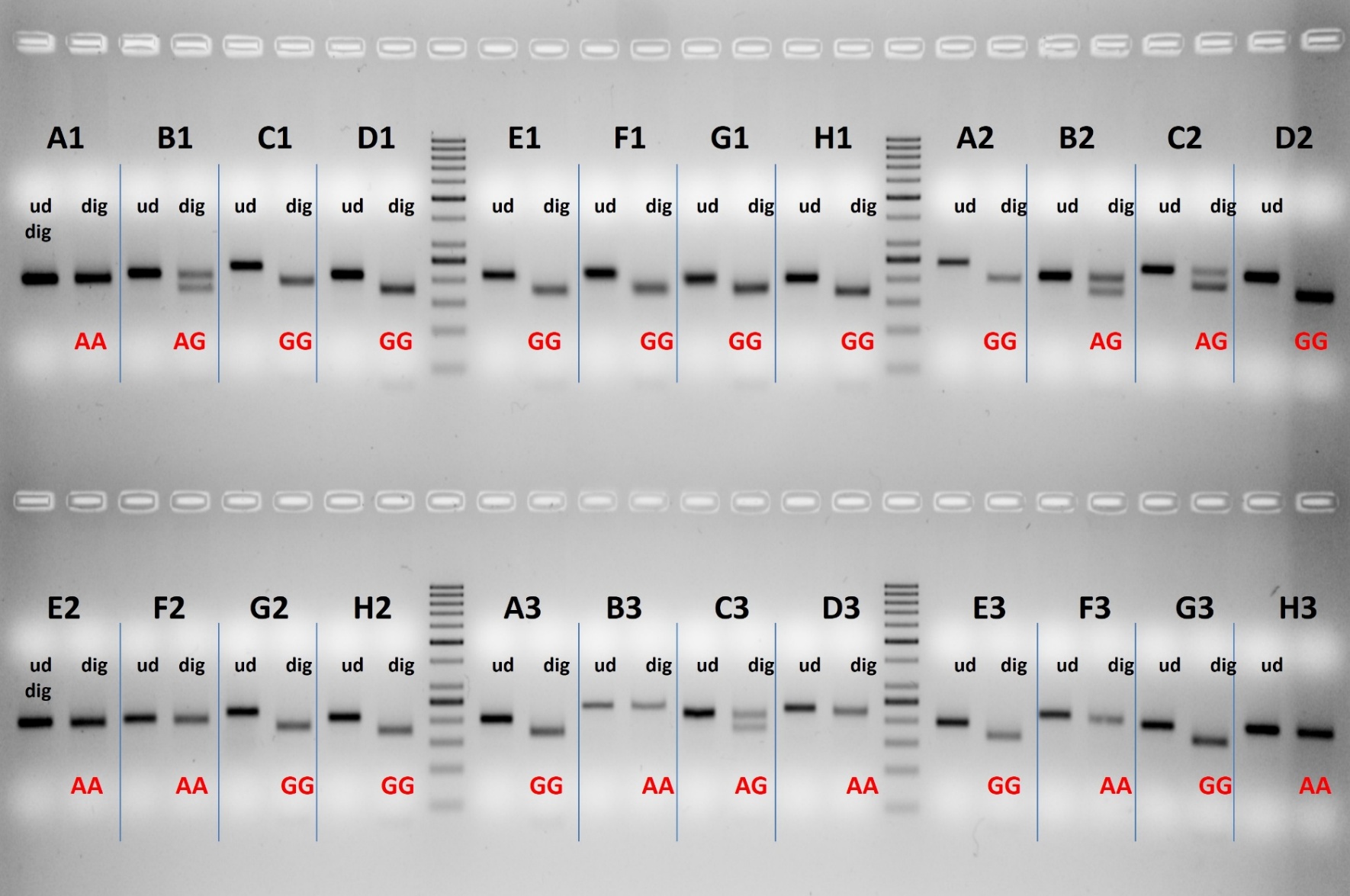

Supplement: S3 File — (Size of undigested PCR products −190 bp; After restriction digestion, AA genotypes showed a single band of 190 bp; GG genotypes showed a single band of 160 bp; AG genotypes showed double bands of 190 and 160 bp; A 50 bp standard size marker ladder was used for resolving band size). (DOCX) [file pone.0337392.s003.docx]
