# Supplementary material for: Development and validation of a simple, cost-effective competitive allele-specific PCR assay for largescale screening and detection of FecB mutation in sheep
Source: PLoS One. 2025 Dec 2;20(12):e0337392. doi: 10.1371/journal.pone.0337392 (PMC12671827; doi:10.1371/journal.pone.0337392)

S3 File. Raw images of PCR-RFLP genotyping of BMPR1B locus using restriction enzyme Ava II

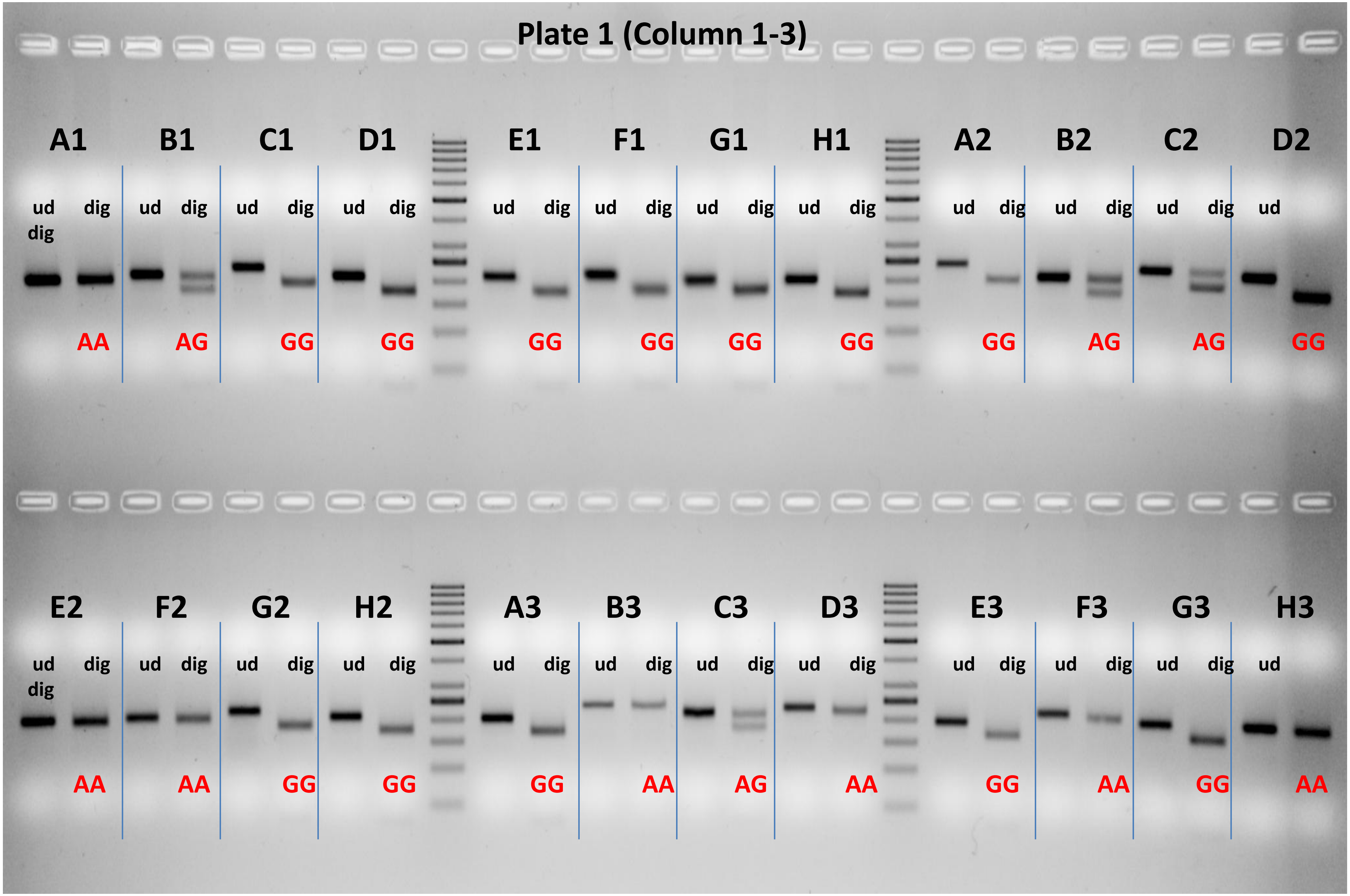

Plate 1 (Column 4-6)

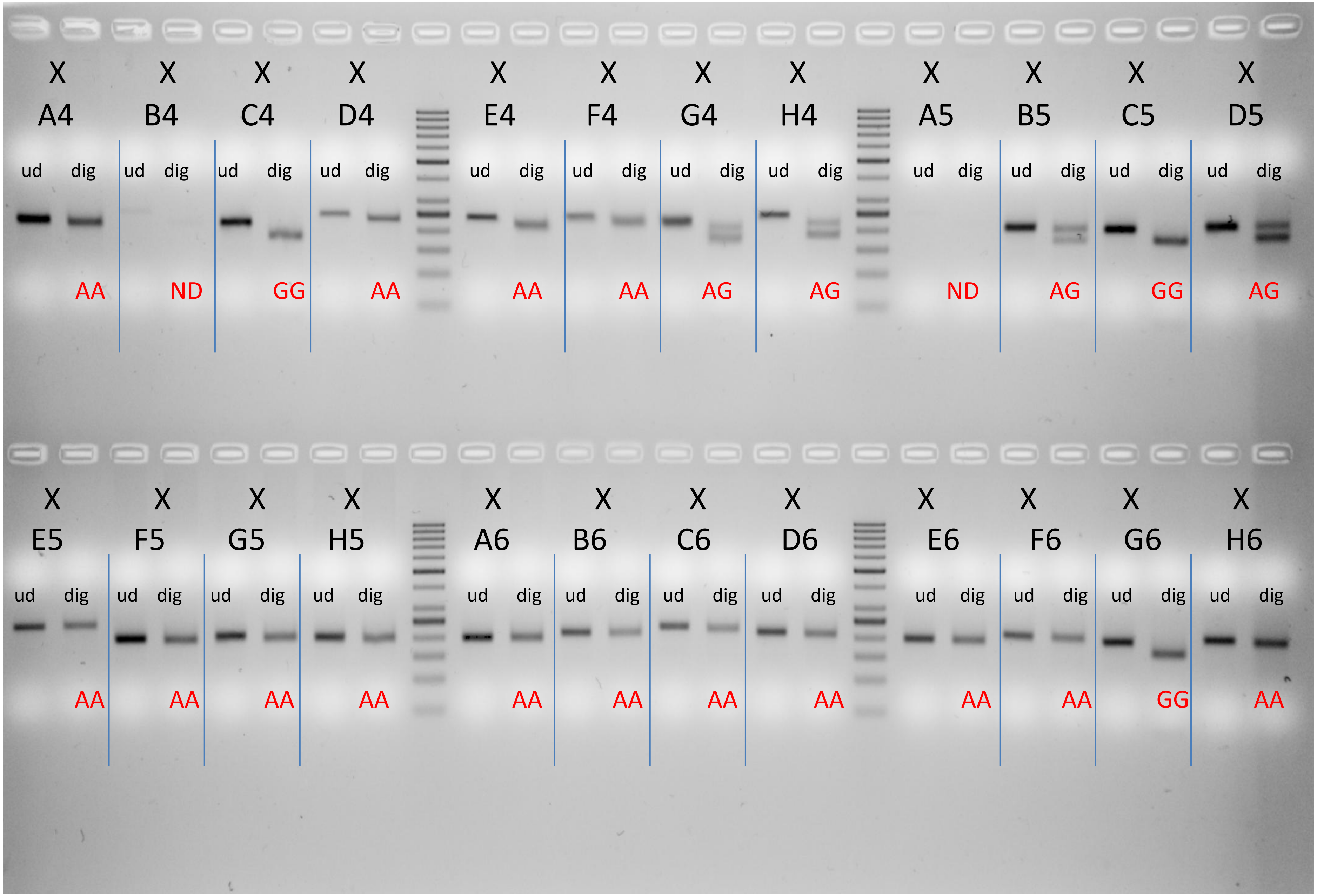

Plate 1 (Column 7-9)

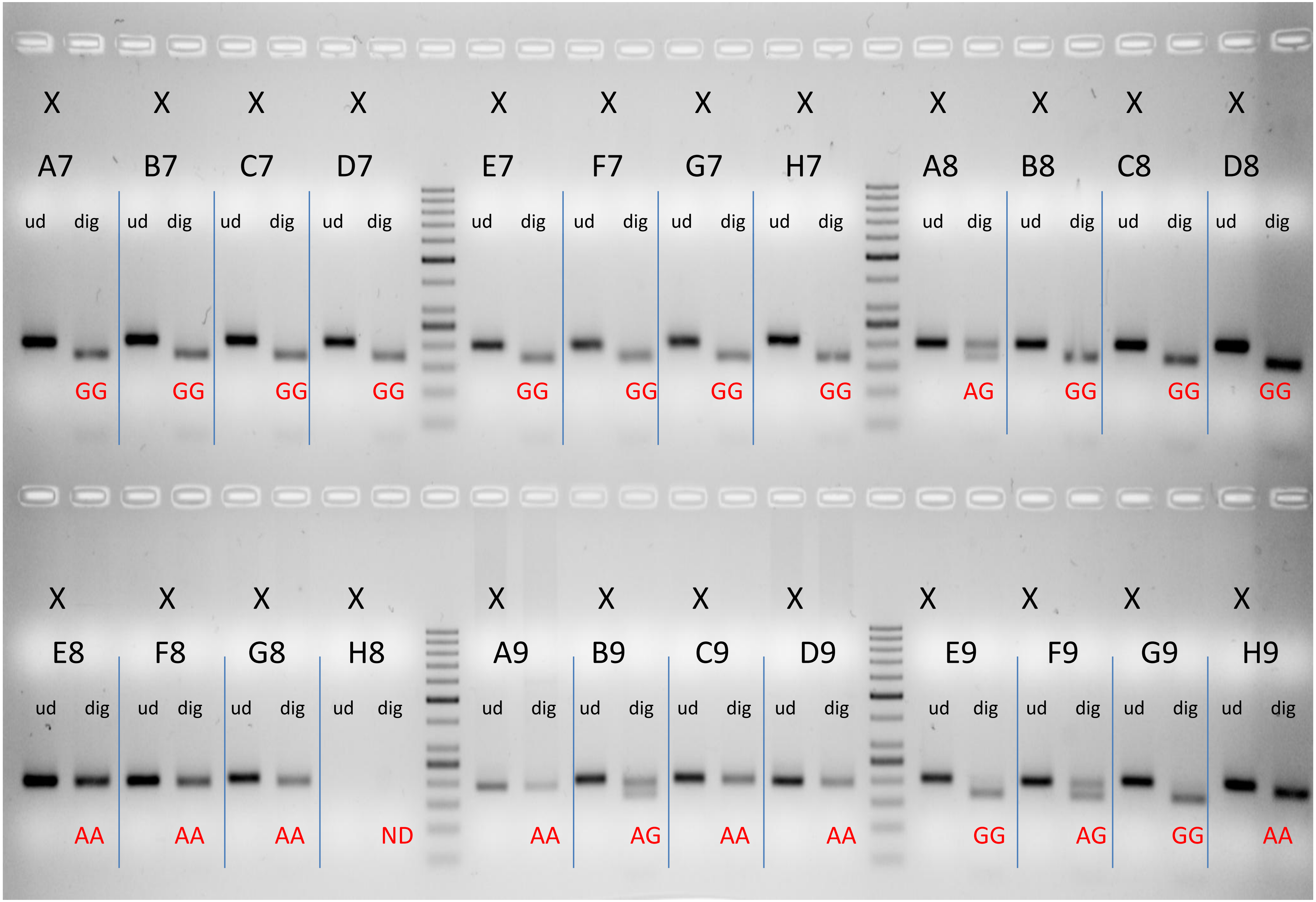

Plate 1 (Column 10-12)

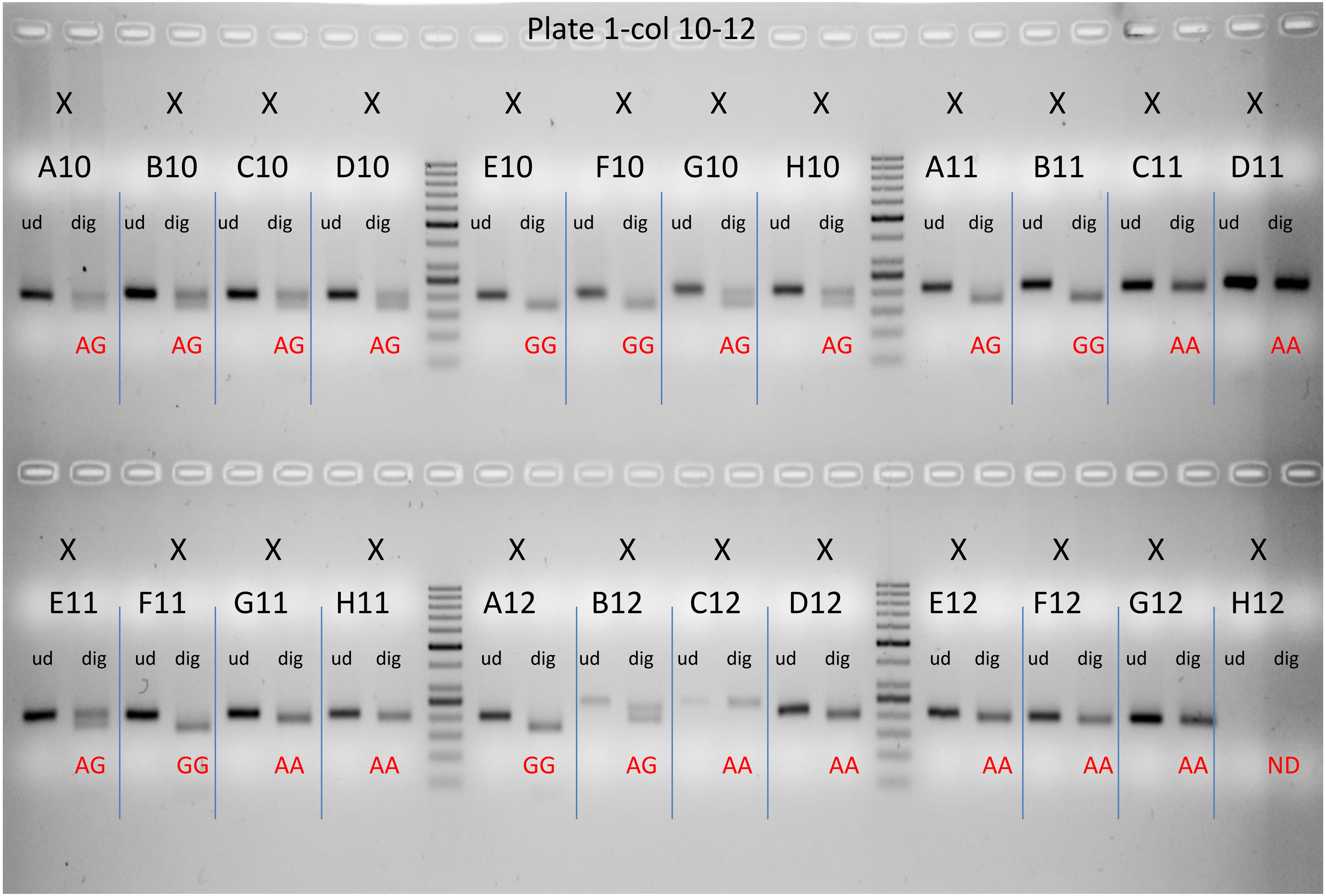

Plate 2 (Column 1-3)

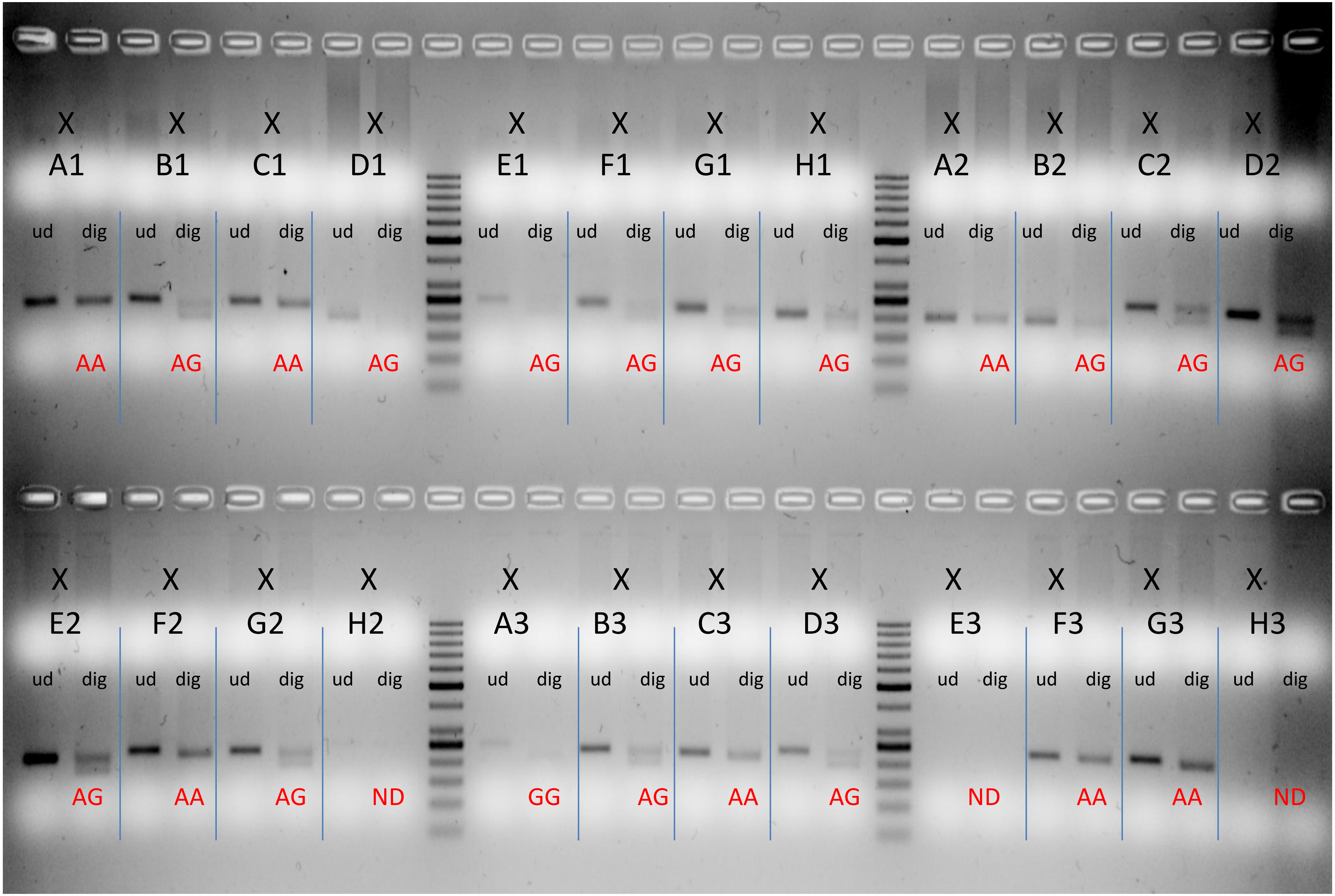

Plate 2 (Column 4-6)

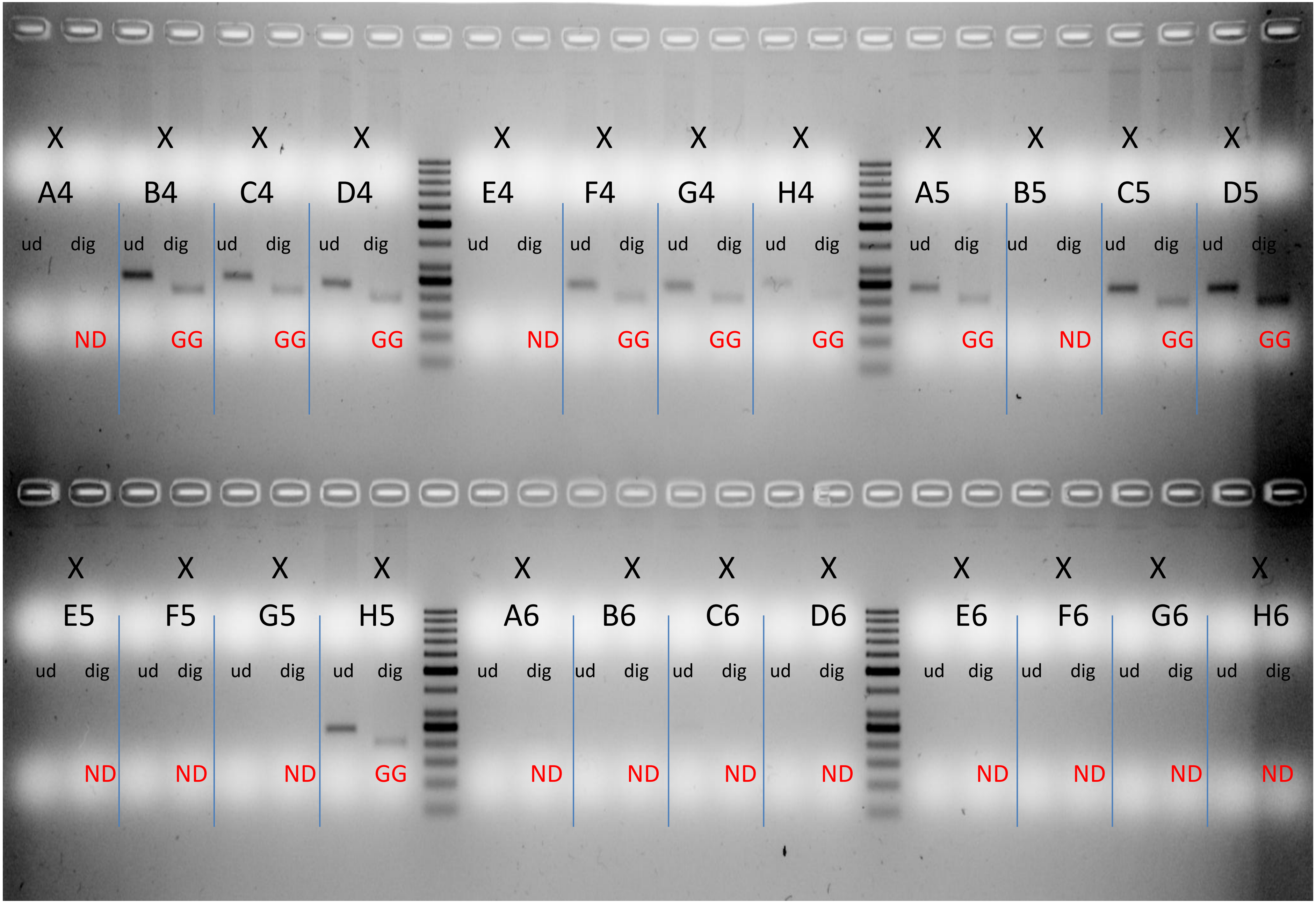

Plate 2 (Column 7-9)

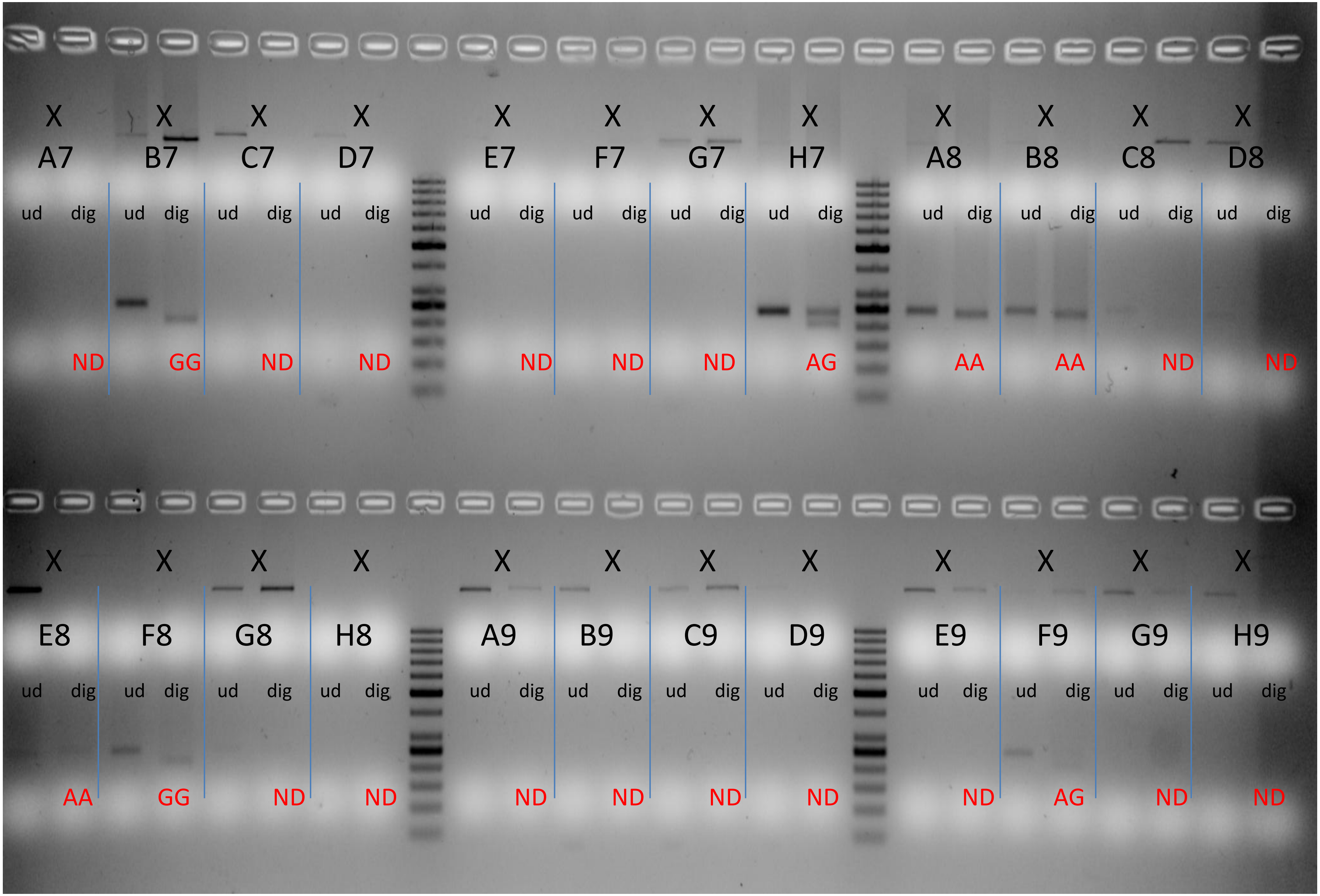

Plate 2 (Column 10-12)

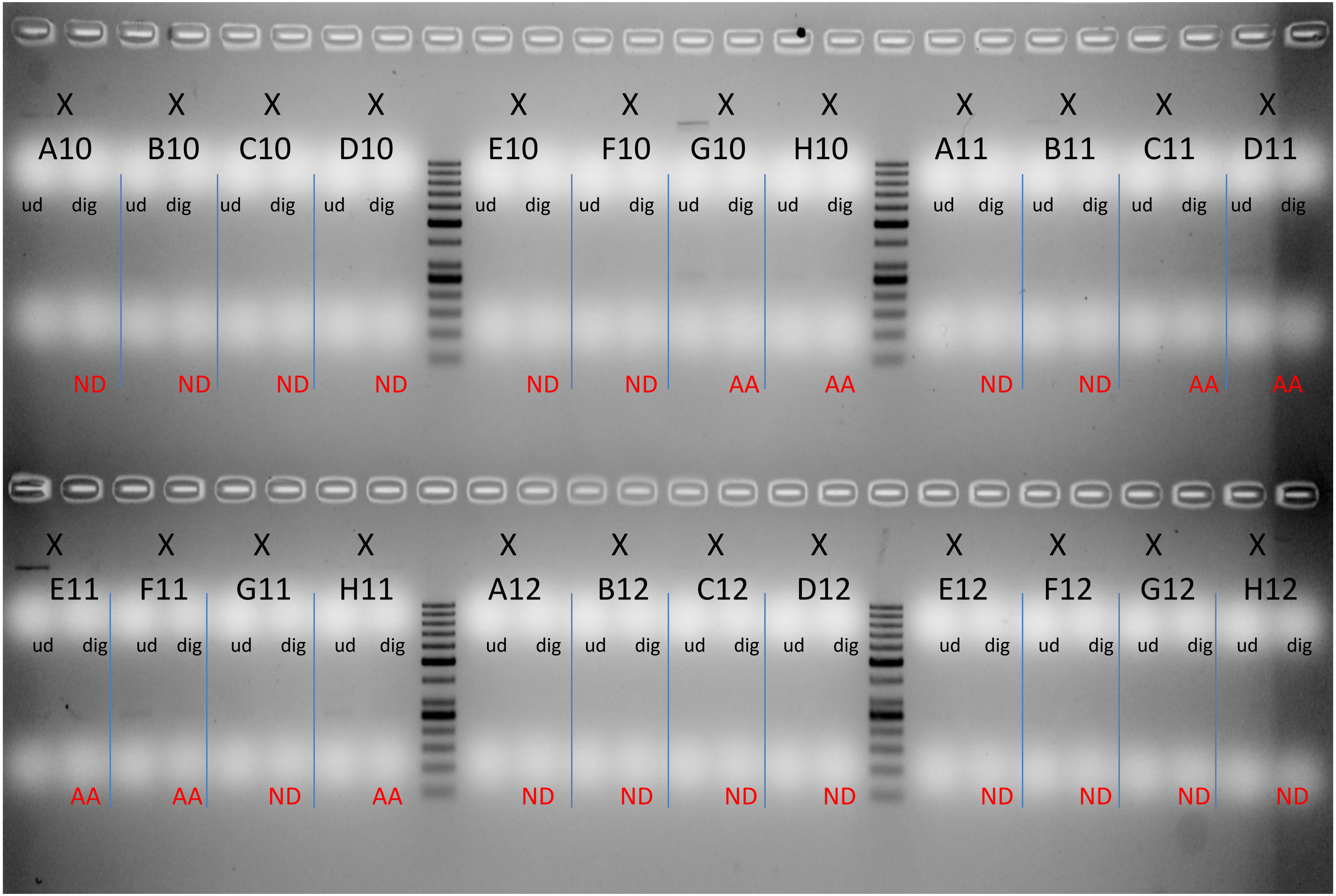

Plate 3 (Column 1-3)

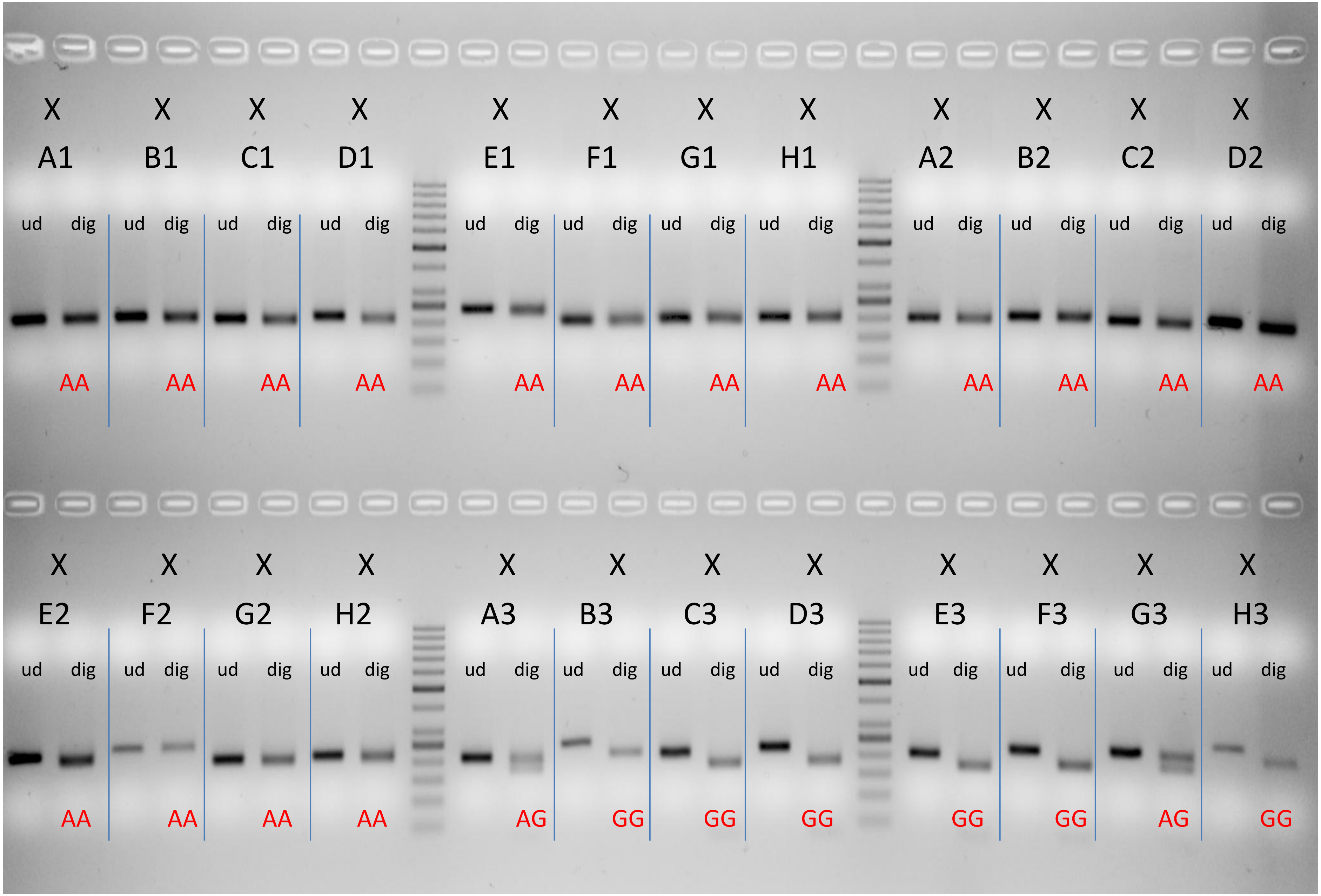

Plate 3 (Column 4-6)

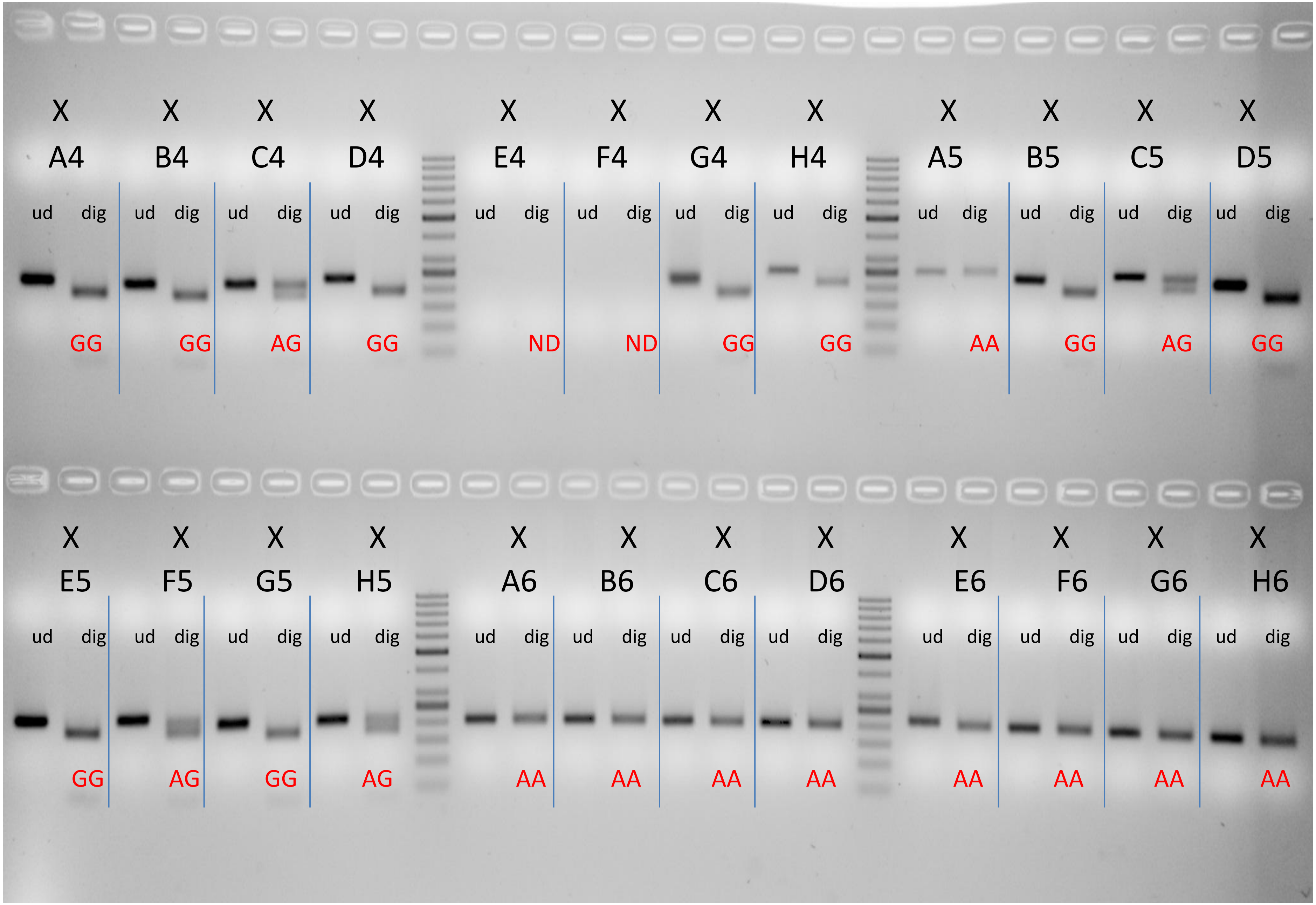

Plate 3 (Column 7-9)

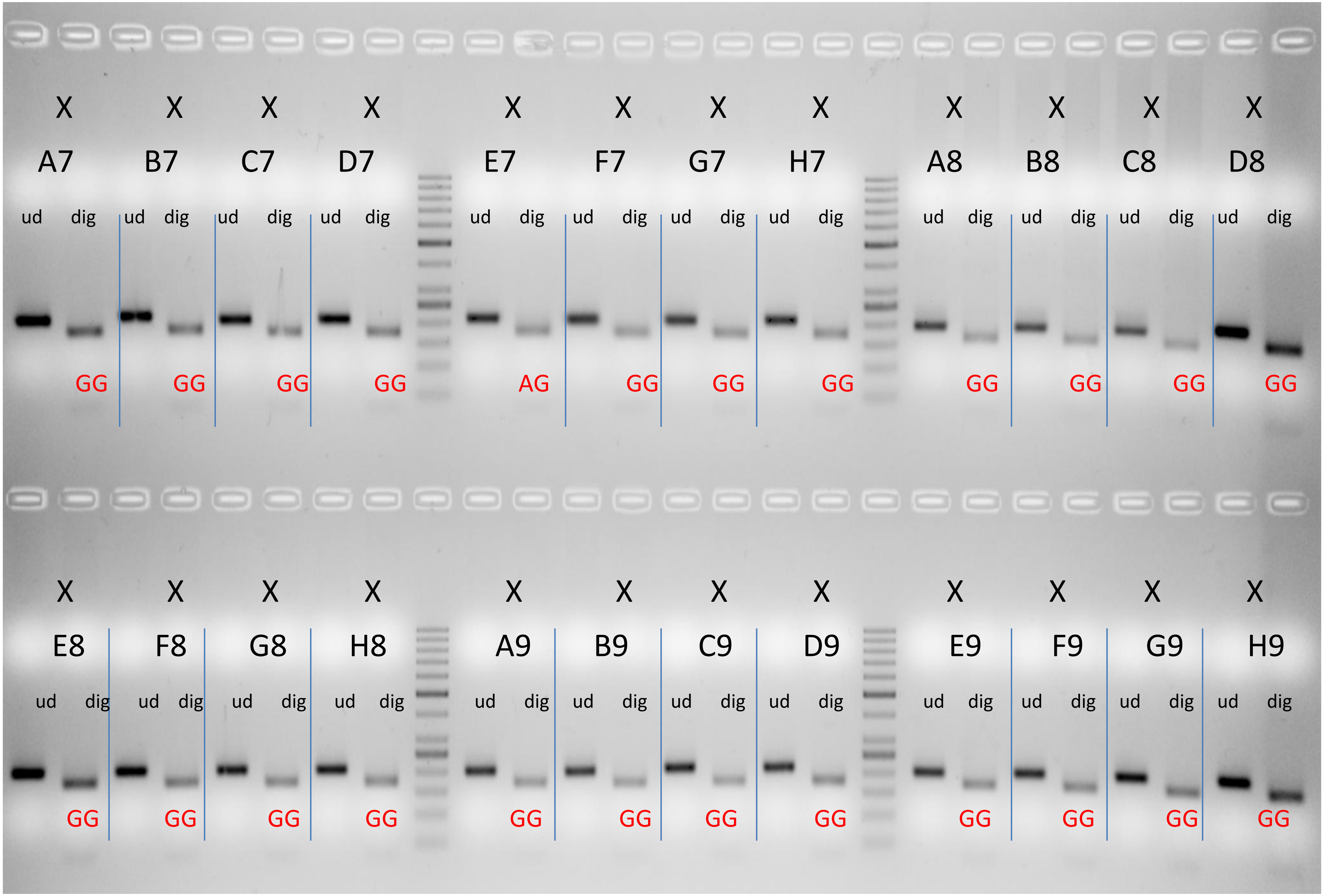

Plate 3 (Column 10-12)

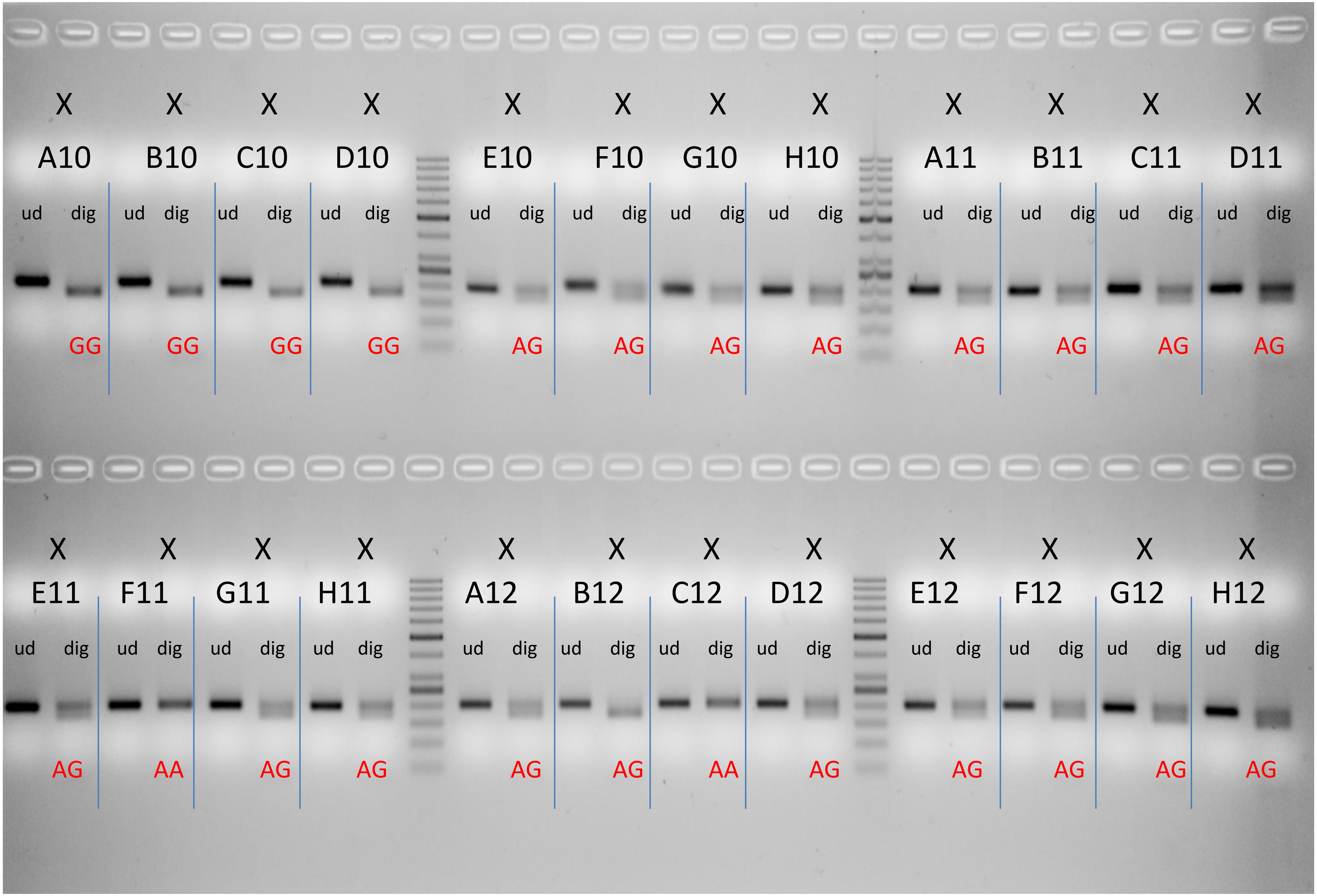

Supplement: S4 File — (PDF) [file pone.0337392.s004.pdf]
